# Assessment of Seismic Performance and Structural Health Monitoring of a Retrofitted Reinforced Concrete Structure with Polyurethane-Based Interventions and Vertical Greenery Systems

**DOI:** 10.3390/polym17233104

**Published:** 2025-11-22

**Authors:** Theodoros Rousakis, Vachan Vanian, Martha Lappa, Adamantis G. Zapris, Ioannis P. Xynopoulos, Maristella E. Voutetaki, Stefanos Kellis, George M. Sapidis, Maria C. Naoum, Nikos A. Papadopoulos, Violetta K. Kytinou, Martha Karabini, Athanasia Thomoglou, Constantin E. Chalioris

**Affiliations:** 1 Department of Civil Engineering, Democritus University of Thrace, 67100 Xanthi, Greece; mlappa@civil.duth.gr (M.L.); azapris@civil.duth.gr (A.G.Z.); ixynopou@civil.duth.gr (I.P.X.); skellis@civil.duth.gr (S.K.); gsapidis@civil.duth.gr (G.M.S.); mnaoum@civil.duth.gr (M.C.N.); nikpapad@civil.duth.gr (N.A.P.); vkytinou@civil.duth.gr (V.K.K.); mkarampi@civil.duth.gr (M.K.); athomogl@civil.duth.gr (A.T.); chaliori@civil.duth.gr (C.E.C.); 2 Architectural Engineering Department, School of Engineering, Democritus University of Thrace, 67100 Xanthi, Greece; mvouteta@arch.duth.gr

**Keywords:** vertical forest, seismic retrofitting, structural health monitoring (SHM), piezoelectric sensors (PZT), renovation, seismic polymer joint, fiber rope, resilient design

## Abstract

This study examines Phase B of the GREENERGY project focusing on the seismic performance and structural health monitoring of a renovated single-story RC frame with brick masonry infills that received significant strategic structural interventions. The columns were confined with basalt fiber ropes (FR, 4 mm thickness, two layers) in critical regions, the vertical interfaces between infill and concrete were filled with polyurethane PM forming PUFJ (PolyUrethane Flexible Joints), and glass fiber mesh embedded in polyurethane PS was applied as FRPU (Fiber Reinforced PolyUrethane) jacket on the infills. Further, greenery renovations included the attachment of five double-stack concrete planters (each weighing 153 kg) with different support-anchoring configurations and of eight steel frame constructions (40 kg/m^2^) simulating vertical living walls (VLW) with eight different connection methods. The specimen was subjected to progressively increasing earthquake excitation based on the Thessaloniki 1978 earthquake record with peak ground acceleration ranging from EQ0.07 g to EQ1.40 g. Comprehensive instrumentation included twelve accelerometers, eight draw wire sensors, twenty-two strain gauges, and a network of sixty-one PZTs utilizing the EMI (Electromechanical Impedance) technique. Results demonstrated that the structure sustained extremely high displacement drift levels of 2.62% at EQ1.40 g while maintaining structural integrity and avoiding collapse. The PUFJ and FRPU systems maintained their integrity throughout all excitations, with limited FRPU fracture only locally at extreme crushing zones of two opposite bottom bricks. Columns’ longitudinal reinforcement entered yielding and strain hardening at top and bottom critical regions provided the FR confinement. VLW frames exhibited equally remarkably resilient performance, avoiding collapse despite local anchor degradation in some investigated cases. The planter performance varied significantly, yet avoiding overturning in all cases. Steel rod anchored planter demonstrated superior performance while simply supported configurations on polyurethane pads exhibited significant rocking and base sliding displacement of ±4 cm at maximum intensity. PZT structural health monitoring (SHM) sensors successfully tracked damage progression. RMSD indices of PZT recordings provided quantifiable damage assessment. Elevated RMSD values corresponded well to visually observed local damages while lower RMSD values in columns 1 and 2 compared with columns 3 and 4 suggested that basalt rope wrapping together with PUFJ and FRPU jacketed infills in two directions could restrict concrete core disintegration more effectively. The experiments validate the advanced structural interventions and vertical forest renovations, ensuring human life protection during successive extreme EQ excitations of deficient existing building stock.

## 1. Introduction

Vertical forest buildings integrate substantial vegetation on facades and rooftops, adding mass and eccentric loads that create seismic challenges for existing structures [1]. Older RC buildings in Mediterranean regions have deficiencies typical of pre-modern construction—sparse column stirrups, inadequate beam-column joints, strong beams or slabs and weak columns, and brick masonry infills vulnerable to fragile cracking, early debonding from concrete frames and potential collapse [2]. The brick infills may increase initial stiffness of the building but stress concentrations at brick–concrete member interfaces may lead to early bond disintegration or infill collapse or even form short-column effects during EQ excitations that are detrimental for the global structure performance. Common shear-related diagonal cracking of infills during earthquakes further raises the uncertainty of post-damage infill–rc frame interaction and potential partial collapses (in and out of plane). The traditional last row of inclined wedged bricks forms a weak infill region, as well as frequently met infill openings because of doors and windows or glazing [3,4,5]. Adding vertical forest renovations (i.e., planters at 600 kg/m^2^, vertical living walls at 40 kg/m^2^) requires addressing these existing weaknesses in as-built structure besides safely anchoring the greenery systems under seismic loading [6,7,8].

Nowadays, a variety of composite materials may offer alternative, more advantageous, natural, recyclable, reusable, fast-activated (no or short curing time), corrosion-free, lightweight, highly deformable, thermal-insulation compatible, elevated temperature-resistant, non-toxic, reversible, and cost-efficient retrofit options that are considered local and do not alter the initial stiffness of primary or infill members; basalt fiber ropes with elastic modulus 69 GPa can confine columns [9,10,11], polyurethane materials with low elastic modulus (4–16 MPa) [12], and high deformability (40–110% strain) [13] can create flexible seismic joints at infill-frame interfaces. Finally, fiber meshes embedded in polyurethane can jacket damaged infills to prevent out-of-plane collapse [13,14]. However, their combined performance in (a) deficient old-type as-built structures, (b) receiving additional mass of vertical forest integration (considered mainly as secondary attached structure) remains largely untested, particularly under (c) progressive seismic loading approaching structural limits. PZT sensors using the EMI technique enable real-time damage detection throughout testing by measuring the electromechanical impedance changes that correlate with structural damage, operating at high frequencies (10–250 kHz) to detect incipient damage before visual evidence appears, with RMSD indices quantifying damage severity [15,16,17,18]. In the study of Voutetaki et al. [16], an experimental investigation on standard 150 mm cubes is carried out for validating the sensors’ measurement range in concrete. The PZT SHM technique has proven effective for concrete elements and steel reinforcement but application on fiber ropes, on infill walls and interfaces, on polyurethane FRP jackets, as well as on vertical forest anchoring connections for early damage detection and human protection is an urgent gap to be identified.

Existing research on seismic retrofitting addresses column confinement, infill strengthening, or structural health monitoring separately, but comprehensive experimental validation of integrated retrofit strategies combined with vertical forest anchoring systems under extreme seismic loading is lacking. Most studies test individual retrofit techniques at moderate intensity levels [19], while the performance of multiple simultaneous interventions [20] (confinement + flexible joints + jacketing + anchored masses) at drift levels approaching 3% remains unexplored. Furthermore, anchoring strategies for roof or balcony-mounted planters and facade-mounted vertical living walls have not been systematically evaluated under progressively increasing seismic excitation intensity to establish performance hierarchies and failure mechanisms [21,22,23]. Shake table testing with incrementally scaled real earthquake records (rather than single-intensity design-level excitations) provides comprehensive data across multiple damage states from elastic response through steel yielding to near-collapse conditions, enabling validation of both retrofit effectiveness and monitoring system sensitivity [13]. Novel urgent need is also the utilization of PZT SHM before and after successive severe earthquake excitations for a very dense network of 61 wireless PZT sensors, the strategy of placement (position, 3d orientation, grouping, and adequate bonding on different materials and surfaces) to maximize damage detection and even the processing time involved to achieve close to real-time practical decision making, coupled with advanced hybrid testing-simulation approaches.

The GREENERGY project examines integration of vertical forest systems attached to seismically deficient RC structures. Phase A characterized the as-built specimen behavior—a single-story RC frame with 130 × 130 mm columns (4Ø8 mm longitudinal bars, Ø5.5/60 mm stirrups), partial and full masonry infills (60 mm thick hollow clay bricks), revealing infill-frame separation, and diagonal cracking at seismic serviceability limit states of 1.13% foundation-top slab drift and of 0.81 of steel yield strain for longitudinal column bars (no steel yielding). Phase B implements comprehensive retrofitting; basalt composite ropes (4 mm thickness, two layers) confine column critical regions, polyurethane PM fills 19–29 mm gaps creating PUFJ at infill-frame interfaces, glass fiber mesh (360 g/m^2^) embedded in polyurethane PS creates FRPU jackets on cracked infills, five double-stack concrete planters (each 153 kg) with five different anchoring configurations simulate roof or balcony greenery, and eight steel frames (40 kg/m^2^) with eight different connection methods simulate vertical living walls.

The current study examines Phase B seismic performance and structural health monitoring of the renovated specimen, focusing on retrofit effectiveness under progressively increasing earthquake excitation (Thessaloniki 1978 record scaled from EQ0.07 g to EQ1.4 g), following a dynamic pushover approach. Further, it focuses on the performance of diverse anchoring systems for vertical forest components, PUFJ and FRPU integrity throughout progressive damage states as well as column behavior with basalt rope confinement through yielding and strain hardening. Finally, it investigates PZT-based damage detection for both conventional structural elements and novel retrofit materials using sixty-one sensors: forty-eight embedded from Phase A and thirteen added for renovation monitoring.

## 2. Methodology

### 2.1. As-Built Specimen

**Figure 1:**
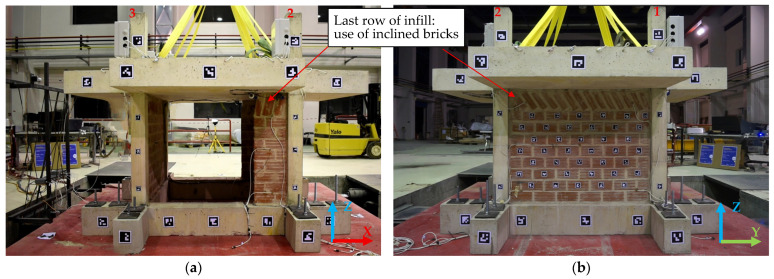

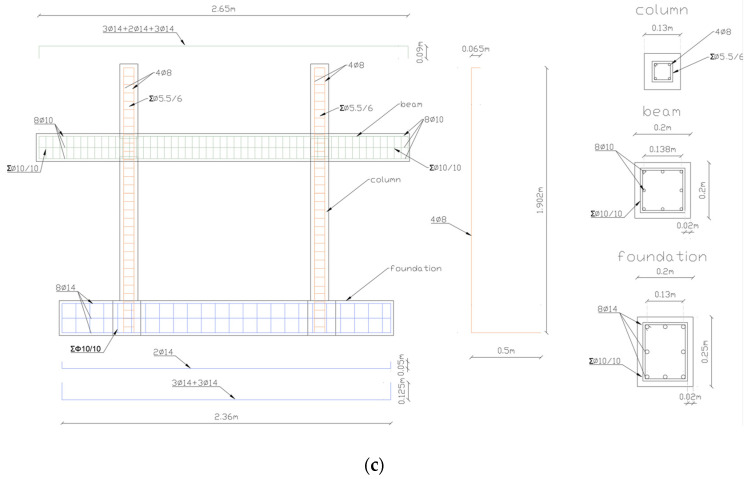
Overview of experimental as-built specimens on the DUTh seismic table: (**a**) frame view showing partial infills, (**b**) frame view showing full infills, and (**c**) reinforcement details. (Adapted from [24]).

The reference structure that underwent structural and greenery interventions is described in detail in the study by [24]. However, for clarity in further notation, the main characteristics of the specimen are provided here as well (see Figure 1). The specimen was deliberately designed with inadequate reinforcement to authentically represent seismically deficient existing building stock. This intentional deficiency is essential for Phase B validation, as the vertical forest retrofit strategy must prove effectiveness on the most vulnerable existing building stock, not on code-compliant structures [24]. The specimen consists of a Reinforced Concrete (RC) frame elevated one story and extended one bay. In other words, it is composed of four rectangular columns (cross-section of 0.13 m by 0.13 m) that are monolithically connected with a system of strong foundation beams (with rectangular cross-section of 0.2 m width by 0.25 m height) that are properly fixed to the seismic-table metallic plate with steel anchors. At the top, the columns are connected with beams of rectangular cross-section 0.2 m by 0.2 m. These beams are characterized as hidden (embedded inside the slab) as they are the same height as the RC slab. The slab extends beyond the rectangular top edges by a length of 0.6 m in the form of cantilevers. These extensions will later serve as the proper holders for the planter interventions. The RC frame in two opposite directions is filled with eccentric partial infills (length of 30 cm and then door opening) of 60 mm thickness that follow a special inclined (wedged) last row of brickwork (see Figure 1a), a typical methodology of older construction in the Mediterranean region. In the other direction (perpendicular to the seismic excitation direction), the frame is filled with the same infill configuration and construction methodology but as a full infill (see Figure 1b). The infills are bonded with classic running bond with mortar bed and head joints of 10 mm thickness for both types of infills. The units for constructing these infills have dimensions of 190 mm in length, 90 mm in height, and 60 mm in thickness. The clay unit bricks consist of voids with holes that are extended in the length direction and constitute approximately 45% of the net surface perpendicular to the length direction. The reinforcement information of the specimen is depicted in Figure 1c.

The reinforcement of the specimen consists of B500C ribbed bars that were used for the longitudinal reinforcement in columns, foundation beams, and hidden beams, while S220 smooth bars were used for the column stirrups to simulate the inadequate transverse reinforcement of older constructions. The columns are reinforced with 4Ø8 mm longitudinal bars and Ø5.5/60 mm stirrups. The foundation beams contain 8Ø14 mm longitudinal bars with Ø10/100 mm stirrups, and the hidden beams have 8Ø10 mm longitudinal bars with Ø10/100 mm stirrups. The slab is reinforced with orthogonal Ø8/100 mm steel meshes at both the top and bottom sides. Standard double steel loops (Ø10 mm hooks) were embedded in the top slab specifically for safe handling and transportation of the specimen.

### 2.2. Renovated Specimen with Vertical Forest

#### 2.2.1. Structural Interventions

**Figure 2:**
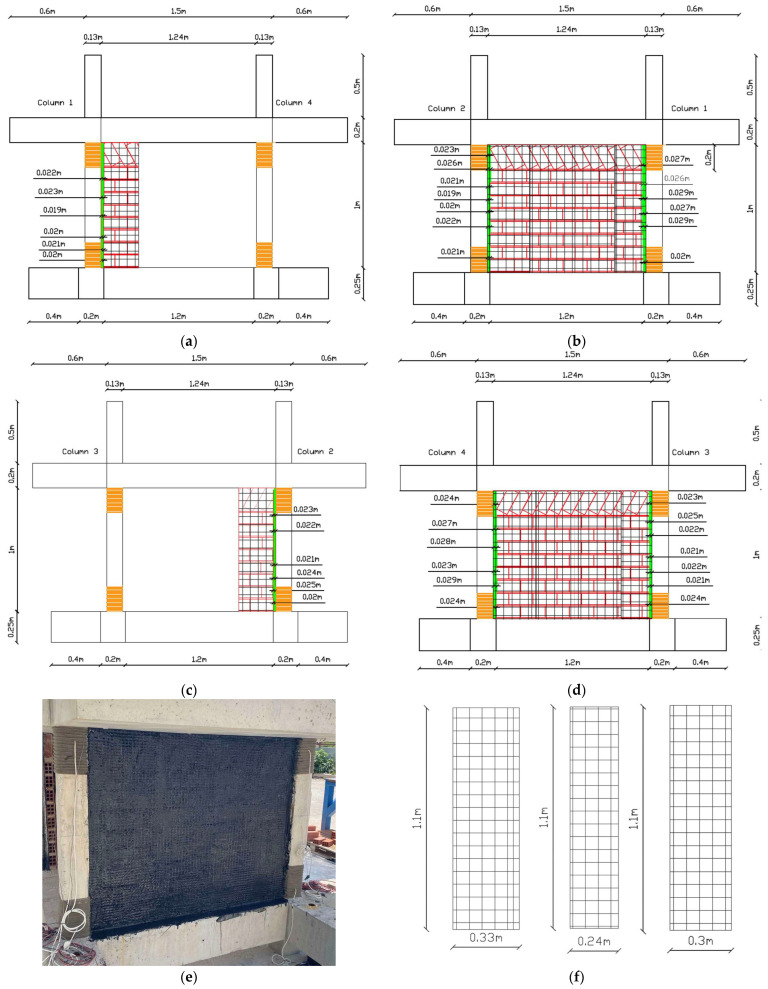
Structural interventions view of (**a**) frame 1-4, (**b**) frame 1-2, (**c**) frame 2-3, (**d**) frame 3-4, (**e**) final experiment frame 2-3 and 3-4, and (**f**) infill jacket mesh part details.

The as-built specimen received significant strategic structural interventions as depicted in Figure 2. The order of application was as follows: columns basalt rope confinement, seismic polymer joint along vertical infill-concrete interfaces, and brick infill external jacket. The implementations are discussed in detail in the following paragraphs.

To properly insert the seismic joint between all vertical interfaces of infills and concrete frame, a vertical gap needed to be created. A maximum 2.9 cm gap was created using an electrical saw to cut all infills vertically at the interfaces. The procedure was relatively demanding as all top and vertical infill boundaries had already been detached from the surrounding concrete frame after the test loading from Phase A and with internal cracks between bricks (11 excitations with maximum peak ground acceleration of EQ1.1 g and 1.13% structure drift). For the partial infill, the gap ranged from 1.9 cm to 2.5 cm and for the full infill, it ranged from 1.9 cm to 2.9 cm. A total of 6 vertical PUFJs were constructed in situ (denoted in Figure 2 with green color at the infill and concrete interfaces), followed by grinding of the external concrete surface to expose aggregates and ensure proper PU bonding. Special care was taken for the absorption of brick dust during infill sawing and cement dust during grinding. The seismic joint gaps provided the adequate space for rounding the column edges (through grinding) in order to receive fiber rope wrapping.

The critical regions of the columns were properly rounded as good practice for properly applying composite confinement material based on guidelines from [9,10]. The corners of the columns were carefully rounded, creating a curvature that ranged from 16 mm to 22 mm based on the available working space. With this curvature, the average perimeter of 52 cm was reduced for each column as follows—Column 1: Bottom perimeter 48.8 cm, Top perimeter 47.4 cm; Column 2: Bottom perimeter 48.6 cm, Top perimeter 48.0 cm; Column 3: Bottom perimeter 48.6 cm, Top perimeter 48.6 cm; and Column 4: Bottom perimeter 48.4 cm, Top perimeter 47.6 cm. The rounding extended at least 20 cm from both the top and bottom regions of the columns. Afterward, the perimeter was properly cleaned with hard brushes to remove any remaining dust from the rounding procedure.

In the next step, continuous basalt fiber rope was wrapped around the rounded external section of the columns (see Figure 2a–d orange color in column top and bottom region). Basalt fiber rope is inert, natural, continuous flexible reinforcement that possesses similar mechanical properties with glass fiber ropes. Further, as already concluded in [9,10,11] (among others) continuous fiber rope wrapping needs no impregnation or bonding polymers for confinement applications. The external confinement provided by this (reused) green, reusable, recyclable, natural, high temperature-resistant, and corrosion-resistant flexible fiber material extended vertically about 200 mm. In total, 2 layers of rope were used. The first layer provided the actual confinement, and the second layer was used for self-anchoring to the columns. The thickness of the basalt ropes was approximately 4 mm. For a mean concrete perimeter of 485 mm, accounting for the rope thickness (resulting in an effective perimeter of 501 mm at the centerline of the rope), the total length of rope used for one layer was approximately 25.1 m (for 50 spirals). The detailed values for each column are provided in Table 1.

Horizontal holes of cut bricks were filled with mortar. To the grinded concrete and exposed brick surfaces, where the seismic joint would be constructed, a primer was applied. The polyurethane PM [13] was applied with the help of wooden molds of 20 cm height. As the clear height of the frame was 100 cm, a total of 5 sequential castings were required by PM pouring. The important part is that the procedure should be completed in a short time as the polyurethane hardens in about half an hour. The final result is denoted in Figure 2 with green color at the infill and concrete interfaces.

The last structural intervention is the use of external jacket on the infills. Fiber mesh (Sika Wrap 350G Grid) was applied to the cracked masonry infills. The mesh, made of glass fiber reinforced polymer, had a weight of 360 g/m^2^. This mesh was impregnated and glued with resin PS (Sika PS), forming FRPU (Fiber Reinforced PolyUrethane) jacket. The application involved cutting the grid into specific dimensions of segments of 1.10 m high by 0.33 m wide and 1.10 m by 0.24 m for full infill and 1.10 m by 0.30 m for partial infill (see Figure 2f). The glass grids were placed on the infill surfaces (had received primer without prior repair of cracks, etc., and then a layer of PS), also covering the polymer joint thickness but not the adjacent columns (in order not to cancel the polymer joint function). Along height, the grid was extended 5 cm from both sides and bonded on the concrete foundation beam and top slab surface (grinded as well). Then, a final layer of PS polyurethane resin is used to cover the voids in the grid. The final results are depicted with black mesh notations on the infill surfaces in Figure 2a–d.

All the structural interventions described above were implemented as part of the comprehensive retrofitting strategy for the Vertical Forest specimen, aiming to enhance seismic performance while integrating green infrastructure elements. The aim was to ensure optimized infill-column interaction and full human protection against full or partial infill or building collapse. Further, it ensures minimum use of non-recyclable materials and maximum use of recyclable, natural materials that can act as damage barriers and all are directly activated. Fiber ropes are fully activated just after wrapping (with no use of resins), PUFJ polymer seismic joints are fully activated just half an hour after casting and FRPUs are fully activated a few hours after jacketing, offering an exceptional emergency intervention method.

#### 2.2.2. Greenery Interventions

**Figure 3:**
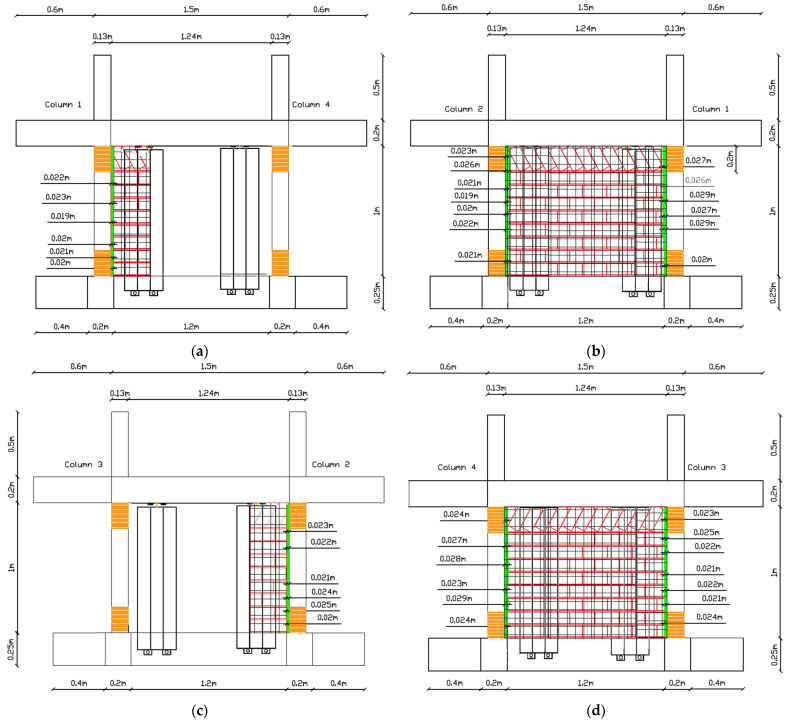

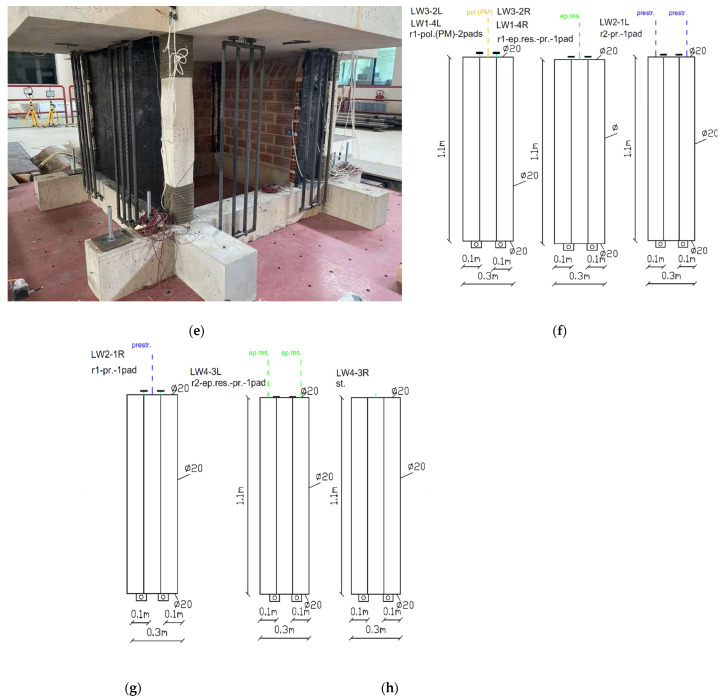
Greenery interventions of simulated living wall, view of (**a**) frame 1-4, (**b**) frame 1-2, (**c**) frame 2-3, (**d**) frame 3-4, (**e**) final experiment frame 2-3 and 3-4 and living wall connection details for (**f**) frame 2-3 and 1-4 (**g**) frame 1-2, and (**h**) frame 3-4.

To simulate the seismic behavior of vertical living walls, special steel frame constructions were made. Each construction was fabricated from vertical steel bars with a diameter of 20 mm (Φ20) along with additional (horizontal) spacers of diameter 10 mm (Φ10) to maintain proper distance from the concrete structure. These steel structures were designed to replicate both the mass and stiffness of vertical living walls construction including the greenery. The weight of each steel structure was 40 kg/m^2^, equivalent to 12 kg per unit, totaling 96 kgs for all eight vertical living walls. The constructions were attached to both the foundation beam (with steel connection) and top slab (with basalt fiber ropes) as shown in Figure 3e–f.

Regarding the attachment of these constructions, eight steel structures were fixed to the specimen during the retrofit phase. Of these, four constructions were placed parallel to the loading direction (two per side—in-plane loading), and four were positioned perpendicular to the loading direction (out-of-plane loading). The bottom connection of each construction to the foundation beam was achieved using the common approach through two steel screw connectors in all cases. For the top connections to the slab, eight different configurations were implemented as follows:

*Out-of-plane—perpendicular to the loading direction (4 different cases):*

1. Common approach with one steel screw connector on the top slab (see Figure 3h right, LW4-3R).
2. Top attachment using two Basalt ropes, anchored through the slab depth. Suitable holes were drilled; pre-tensioning was applied to the ropes with a special metal structure from the upper part of the slab in order to press the steel frame of the vertical living wall against the slab through wedged PM pads. Then, slab holes were filled with epoxy resin (Sikadur^®^-52 Injection LP). After the resin developed its strength, the tensioning device was removed (see Figure 3h left, LW4-3L).
3. Top attachment using two Basalt ropes, anchored through the slab depth. After drilling suitable through holes, pre-tensioning was applied to the ropes with a special metal structure from the upper part of the slab, in order to press the steel frame of the vertical living wall against the slab through wedged PM pads. The tensioning device remained in place throughout the series of testing to function as an active anchoring device (see Figure 3g, LW2-1L).
4. Top attachment using one Basalt rope, anchored through the slab depth. The application procedure was similar to case 3 (Figure 3g right, LW2-1R).

*In-plane—parallel to the loading direction (4 different cases):*

5. Top attachment using one Basalt rope, anchored through the slab depth. A suitable through hole was drilled, then tension was applied to the rope with a special metal structure from the upper part of the slab, in order to press the steel frame of the vertical living wall against the slab through wedged PM pads. Then, the hole was filled with polyurethane resin (Polyurethane PM). After the polyurethane had developed its strength, the tensioning device was removed (Figure 3f left, LW3-2L).
6. Top attachment using one Basalt rope, anchored through the slab depth. A suitable through hole was drilled, then pre-tension was applied to the rope with a special metal structure from the upper part of the slab, in order to press the steel frame of the vertical living wall against the slab through wedged PM pads. Then, the hole was filled with epoxy resin (Sikadur^®^-52 Injection LP). After the resin had developed its strength, the tensioning device was removed (Figure 3f right, LW3-2R).
7. Top attachment using two Basalt ropes, anchored through the slab depth. The application steps were the same as in case 5 (Figure 3f left, LW1-4L).
8. Top attachment using two Basalt ropes, anchored through the slab depth. The application steps were the same as in case 6 (Figure 3f right, LW1-4R).

**Figure 4:**
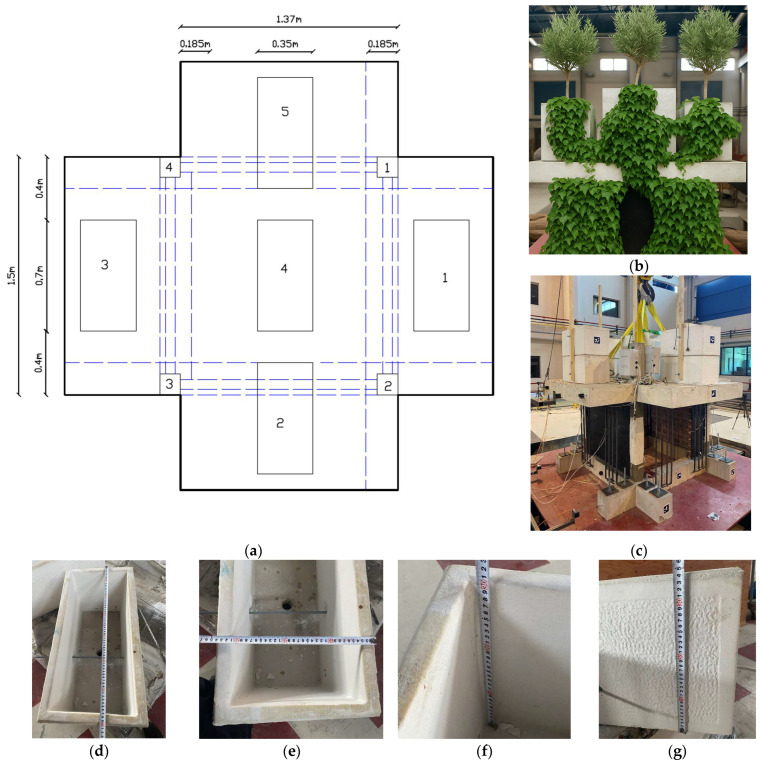
Greenery interventions of planters: (**a**) top view, (**b**) natural representation of view 4-3 of the experimental model, (**c**) perspective view of frame 2-3 and 3-4 of the experimental model, (**d**) and for individual planter the dimensions of (**d**) length, (**e**) width, (**f**) internal height, and (**g**) external height.

As mentioned, all seven novel top supports had polyurethane PM pads of dimensions 40 mm × 40 mm × 5 mm (one or two pieces, depending on the gap of the steel construction from the slab). As depicted in the artistic physical representation in Figure 4b, VLW may serve as compatible green envelope to provide additional protection to the building external surface (control corrosive agents, solar radiation, etc.) while controlling microclimate (shading, humidity, lower temperature extremes, human comfort, etc. [1,7,8]). Advanced renovation may also similarly benefit from suitably designed planters as described in the next section (protection and microclimate).

Additionally, five concrete double-planters were placed on the roof slab (Figure 4a,b) to simulate a greenery mass of 600 kg/m^2^. Each double-planter weighed 153 kg and occupied an area of 0.35 m × 0.7 m (Figure 4a–g). The height of each double-planter was 0.7 m, and the distribution of concrete mass simulated a demanding case that was vulnerable to overturning due to rocking effects during severe earthquake excitation. Furthermore, wooden sticks were attached to the planters to simulate common trees (Figure 4b,c). The total extra mass added to the structure was 153 × 5 + 30 = 795 kg.

For the planter anchoring on the top slab, five different configurations were used:

1. A steel rod passed through the slab and the planter and it was tightened with a nut at the bottom of the slab and at the top of the planter (simulating the anchoring of both the tree rooting system and planter 1, Figure 4a).
2. Two basalt ropes were used as tendons passing through the hole made in the slab. One rope (double) was fully post-tensioned, while the second rope (double) was partially tensioned to serve as a safety rope (planter 2).
3. Two basalt ropes were used as tendons passing through the hole made in the slab. One was fully post-tensioned, while the second was partially tensioned (planter 3).
4. No basalt anchors were used. The planter was simply supported on two opposite PM pads with dimensions 400 mm × 40 mm × 5 mm (planter 4, center).
5. Two basalt ropes were used as tendons that were fully post-tensioned (planter 5).

### 2.3. Materials

#### 2.3.1. Phase A Materials

Two concrete strength categories were used to properly represent older structures. Based on Eurocode 2 [25] classification, C30/37 was used for the foundation beam to provide enhanced bearing capacity with minimum mass, while C20/25 was utilized in the columns, column extensions, and top slab. The concrete structure was cast in the DUTh laboratory using conventional materials with a maximum aggregate size of 16 mm. Quality control involved casting twelve cylindrical specimens (150 mm diameter × 300 mm height) following EN 12390-3 and 6 [26,27] standards. Compressive strength testing revealed the foundation tie beams achieving 36.76 MPa (COV = 2.5%, ready mixed concrete, Alexandros ATEBE), the slab elements 31.63 MPa (COV = 2.9%, ready mixed concrete), and the columns 23.52 MPa (COV = 9.7%, mixed in situ in DUTh lab). The lower column strength aligns with the design intention to simulate deficient older structures.

The reinforcement replicated conventional building methods prevalent in seismic Mediterranean regions. B500C ribbed bars were employed for all longitudinal reinforcements, while S220 smooth bars were used for column stirrups to simulate the sparse transverse reinforcement characteristic of pre-Eurocode construction. The reinforcement layout included 4Ø8 mm longitudinal bars with Ø5.5/60 mm stirrups in columns and 8Ø14 mm longitudinal bars with Ø10/100 mm stirrups in foundation beams. Hidden beams contained 8Ø10 mm bars with Ø10/100 mm stirrups, while the slab was reinforced with Ø8/100 mm bars in both layers. The total steel weight was 390.3 kg. Tensile testing of the B500C steel provided a yield strength of 564 MPa, ultimate tensile strength of 663 MPa, yield strain of 0.289%, ultimate strain of 14.3%, and elastic modulus of 195,156 MPa.

Following the Mediterranean architectural custom, hollow clay bricks (KEBE S.A., Kilkis, Greece) with dimensions of 60 × 90 × 190 mm, 45% void percentage, and 1.2 kg weight were used for masonry infills. The mortar was purposefully created with low strength using lime-cement mortar of 10 mm thickness to simulate traditional building methods.

#### 2.3.2. Phase B Retrofit Materials

The renovation of the specimen in Phase B involved the strategic application of advanced composite materials and polymer-based systems. The retrofit materials were selected to provide enhanced seismic performance while maintaining compatibility with the existing concrete and infill structure, integrating the green protective envelope.

The polyurethane flexible joints were constructed using Sika PM, a flexible polyurethane system designed to accommodate seismic deformations at the interface between the concrete frame and masonry infills. The material exhibited an elastic modulus of 4 MPa and a strength of 1.4 MPa. Tensile testing revealed an ultimate elongation of 110%, demonstrating significant ductility and deformation capacity. This material was poured into the gaps created between columns and masonry infills to provide a flexible seismic joint that could accommodate differential movements during seismic excitation.

The fiber-reinforced polyurethane system utilized Sika PS, which provided enhanced mechanical properties compared to the flexible PUFJ material. The FRPU exhibited an elastic modulus of 16 MPa and a strength of 2.5 MPa, with an ultimate elongation of 40% in tensile tests. This material served as the matrix for the glass fiber mesh reinforcement system and provided additional structural capacity to the retrofitted elements.

Sika Wrap 350G Grid, a glass fiber-reinforced polymer mesh, was used to provide tensile reinforcement to the PS matrix. The mesh had a real weight of 360 g/m^2^ and exhibited significantly high mechanical properties, with an elastic modulus of 80,000 MPa, a strength of 2600 MPa, and ultimate elongation of 4%. The mesh was impregnated and glued with the PS resin to ensure proper composite action between the fibers and the polymer matrix.

Braided basalt fiber rope (Juan Gili S.L., Barcelona, Spain) was utilized for the external confinement of columns at critical regions. The basalt rope exhibited a tensile modulus of elasticity of 69.3 GPa and an ultimate stress of 1282 MPa for its fibers. According to the manufacturer’s specifications, the elongation at failure of the fibers was 1.85%. The rope had an approximate thickness of 4 mm and was wrapped in two layers around the column perimeter by hand, with the first layer providing the actual confinement and the second layer serving for self-anchoring purposes. No resin was used for impregnation or bonding, or anchoring.

Primer resins were applied to the surfaces prior to the application of PM and PS resins to ensure proper bonding. Additionally, polyurethane PM pads with dimensions of 40 mm × 40 mm × 5 mm were used at various locations to accommodate gaps and provide cushioning at connection points between steel structures and the concrete slab for the vertical living walls.

### 2.4. Instrumentations

The instrumentation from Phase A is described in detail in study [24]. Here, a brief description is provided additionally to the detailed new sensor installed in this phase (Phase B).

**Figure 5:**
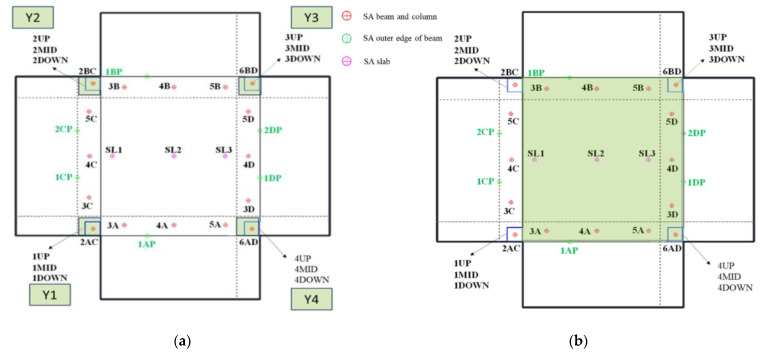

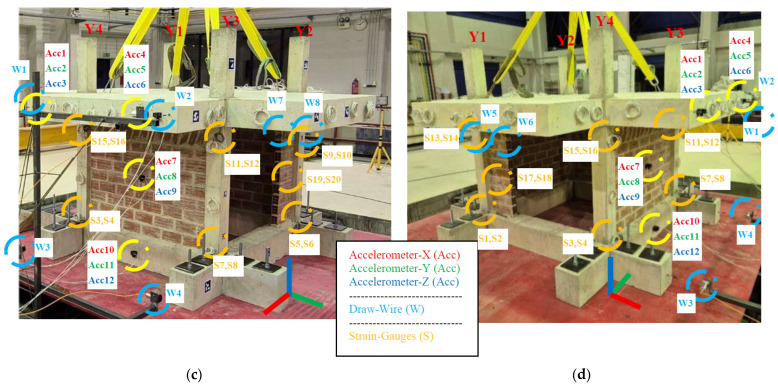
Position of the sensors from Phase A: PZT (**a**) inside the columns (**b**) inside the slab, and (**c**,**d**) draw-wires, accelerometers, and strain-gauges. Note: with letter Y we denote the columns.

In Phase A, the specimen was comprehensively instrumented to capture the complete dynamic response during seismic excitation. The instrumentation included the following (see Figure 5): twelve uniaxial accelerometers arranged in four triaxial clusters to record three-dimensional movement at the slab level, infill center, and foundation level; eight draw wire sensors to capture horizontal displacement data, with four monitoring lateral drift at the upper slab and foundation beam, and four positioned diagonally across partial infill walls to track extension and compression; twenty strain gauges installed on longitudinal reinforcement bars at expected peak moment locations, potential plastic hinge zones, and beam-column joints, as well as on infill panel surfaces; and forty-eight piezoelectric transducers (PZTs) strategically distributed throughout the structure, with thirty-seven embedded as smart aggregates within concrete elements and eleven externally bonded to masonry surfaces. Further details on the specification, installation, function, and output of the PZT network measurements are presented in [24].

For Phase B, the instrumentation was expanded to monitor the retrofitted elements and the structural behavior of the renovated structure. The same 12 uniaxial accelerometers (ACC) remained positioned at four locations (identical to the instrumentation of the as-built specimen). At each location, three accelerometers captured the acceleration components in three directions, covering both in-plane and out-of-plane motions at the center of the full infill walls, as well as accelerations of the top slab and the bottom foundation beam. Additionally, the same eight draw wire sensors (W) continued to monitor the horizontal displacements of the foundation beams and of the top slab. These sensors measure linear displacement through a highly flexible, yet tensioned steel wire connected to a cable drum, which provides an output signal proportional to displacement.

In addition to the already installed 20 strain gauges in the as-built structure, 2 more strain gauges were placed on the polyurethane PM at the PUFJ joint. The joint is located between column 1 and the partial brick infill, while the 2 strain gauges were placed on the PUFJ surface (opposite to each other: internal–external face), 5 cm under the top slab along in-plane direction of excitation (Figure 5, X) to measure in-plane compressive and tensile strain, bringing the total of strain gauges to 22.

**Figure 6:**
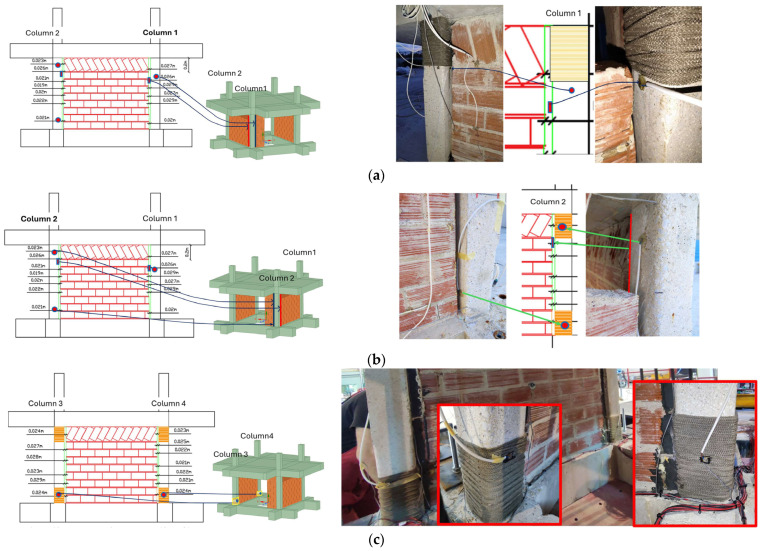

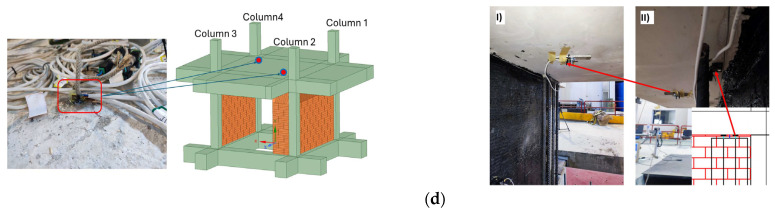
Greenery interventions of new PZT installed (**a**) at the surface of RC column 1, (**b**) at the surface of column 2 and at the surface of the infill inside the polyurethane., (**c**) on Basalt ropes using resin at the bottom of columns 3 and 4, and (**d**) on Basalt ropes using resin on the roof and on the anchoring of metal structures for the (I) planter and (II) Living wall structure.

Furthermore, PZTs were installed as patches at specific locations to monitor the health of the retrofits and the connection (anchoring) of the structures created by the presence of the vertical forest. More specifically, 13 extra PZTs were placed on the renovated structure:

- 1 on concrete column wrapped with basalt rope at the top of column 1 (Figure 6a),
- 2 on concrete column wrapped with basalt rope at the top and the bottom of column 2 (Figure 6b),
- 1 at the surface of the infill connected to column 2, inside the polyurethane,
- 1 at the surface on the infill connected to column 1,
- 1 on the basalt rope at the bottom of column 3 (Figure 6c),
- 1 on the basalt rope at the bottom of column 4 (Figure 6c),
- 2 on the surface of the roof (Figure 6d),
- 2 on the anchoring metal structures, 1 at the planter’s, and 1 at the living wall’s (Figure 6d).

During the application of basalt rope reinforcement in two layers around the top and bottom of columns to improve structural performance, special care was taken to keep the pre-installed PZT wires safe and intact. The basalt rope was tightly coiled with consistent tension to ensure full contact without affecting the PZT functionality. This method provided effective reinforcement while preserving the integrity of the PZT wiring. The positions of PZT patches on the renovated structure are illustrated in Figure 6a–d.

### 2.5. Loading Protocol

**Figure 7:**
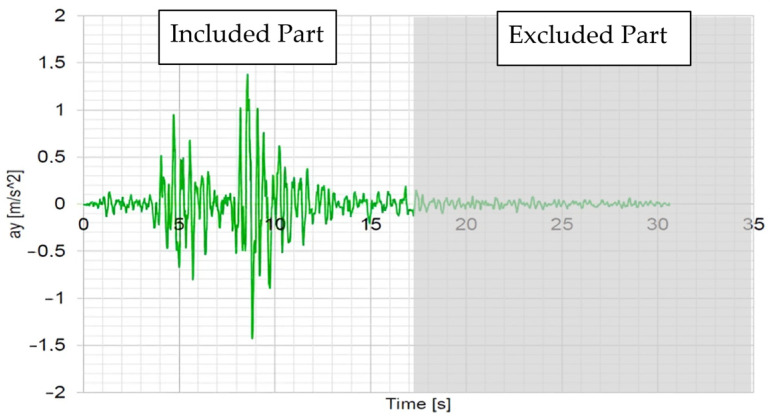
Seismic excitation of original Thessaloniki 1978 earthquake (ay component).

The seismic excitation protocol was based on the record of the Thessaloniki (Volvi) earthquake (see Figure 7), which was one of the most important in modern Greek history and occurred at 23:03 local time on 20 June 1978. The experimental program used a methodological approach of gradually increased intensity [13] to monitor the renovated RC frame response and evaluate the effectiveness of the retrofit interventions, similar to a dynamic pushover approach.

Two types of seismic protocols were employed: (a) low-intensity white noise excitations (0.05 g or 0.08 g depending on the testing stage) to determine the structure’s fundamental dynamic properties and (b) scaled Thessaloniki record from EQ0.07 g to EQ1.4 g at their peak amplitude. For the complete protocol implementation, see Table 2. This was implemented so that the testing procedure could remain manageable while comprehensively assessing the renovated specimen’s behavior under increasing seismic demands. The objective was to evaluate the performance of the retrofit interventions and the structural behavior of vertical living wall and planter constructions under severe seismic excitation.

An important aspect of the Phase B testing protocol was the evaluation of the vertical living wall attachments. The first two white noise tests (ST1 and ST2) were performed to compare the structural response without and with bottom fixing of the vertical living walls, respectively. All subsequent excitations maintained the bottom attachments of the vertical living walls fixed to properly simulate their actual connection conditions.

## 3. Results and Discussion

### 3.1. Acceleration Results

The dynamic acceleration response characteristics of the renovated single-story reinforced concrete structural frame with retrofitted masonry brick infill wall assemblies were comprehensively monitored through a network of twelve precision accelerometers positioned at strategically selected measurement locations throughout the test specimen. The structural system was subjected to fourteen progressively increasing earthquake excitation intensity levels based on the recorded seismic ground motion from the Thessaloniki 1978 earthquake event, specifically with peak ground acceleration amplitudes ranging from EQ0.07 g to EQ1.4 g loading intensity applied through the shake table testing apparatus. The measured peak ground acceleration (PGA) values recorded at the foundation center location by accelerometer ACC10 were 0.07 g, 0.09 g, 0.14 g, 0.21 g, 0.36 g, 0.58 g, 0.63 g, 0.72 g, 0.88 g, 1.33 g, 1.87 g, and 2.56 g for the respective earthquake loading intensities of seismic tests numbers 3, 4, 6, 7, 9, 11, 13, 15, 17, 19, 23, and 25 (ST3, ST4, ST6, ST7, ST9, ST11, ST13, ST15, ST17, ST19, ST23, and ST25), while the corresponding peak floor acceleration (PFA) responses measured at the upper slab level through accelerometers ACC1 and ACC4 reached maximum values of 0.12 g, 0.18 g, 0.22 g, 0.34 g, 0.53 g, 0.77 g, 0.91 g, 1.23 g, 1.31 g, 1.66 g, 1.80 g, and 1.80 g, respectively. The calculated floor-to-foundation acceleration amplification factors (FAF) demonstrated a distinct behavioral trend, namely initially increasing from 1.71 during the lowest intensity seismic excitation to 2.00 at EQ0.1 g loading intensity, then gradually decreasing through progressive damage stages, reaching 1.24 at EQ1.1 g excitation, and finally dropping below unity to 0.96 at EQ1.3 g (2) and 0.71 at the maximum EQ1.4 g excitation level (seismic test number 25), indicating severe structural stiffness degradation and highly nonlinear dynamic response behavior in the renovated infilled reinforced concrete frame system.

**Figure 8:**
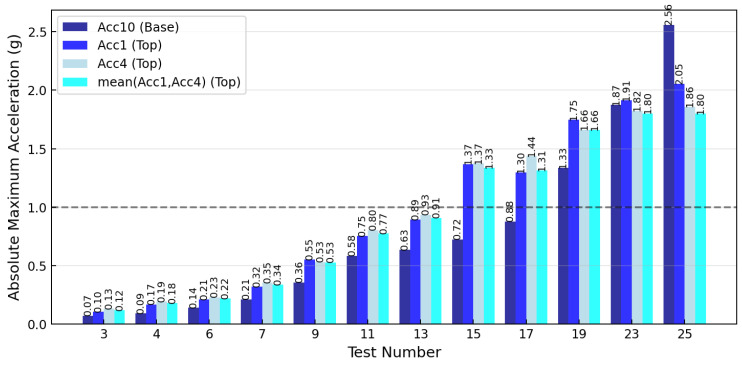
Progressive increase in acceleration (seismic load cases).

During the white noise excitations conducted before and after each earthquake test, additional insights into the dynamic characteristics of the structural system were provided. The white noise testing procedures, performed at consistent 0.05 g or 0.08 g input loading intensities, showed floor acceleration amplification factors ranging from 1.75 to 4.00, with the maximum amplification occurring after the final 0.2 g earthquake loading test (seismic test number 28). The pronounced dynamic amplification during white noise excitations progressively increased from 1.75 before the first excitation to 2.50–3.33 during intermediate testing stages, reaching 3.80 before the repeated 0.2 g excitation and finally 4.00 after the complete testing sequence. This elevated amplification during white noise testing indicated significant changes in the structural stiffness and damping characteristics of the renovated system, which were correlated with the progressive damage accumulation in infill walls, yielding of column reinforcement, and anchor degradation of vertical forest components. The sustained high amplification factors throughout the testing sequence confirmed gradual damage accumulation inside concrete, steel, and brick infills leading to permanent changes to the dynamic properties of the structural system, yet desirable, because of the innovative retrofit interventions. Detailed information is depicted in Figure 8 and Table 3. Herein, it should be reminded that the as-built structure had already suffered the eleven severe excitations of the phase A.

The acceleration response behavior of the masonry infill wall assemblies exhibited particularly interesting dynamic characteristics, with accelerometer ACC7 positioned at the central location of the full infill wall assembly recording dramatic increases in out-of-plane acceleration amplitudes. During seismic test number 15 (ST15) at 0.74 g earthquake loading intensity, this accelerometer registered 1.19 g peak acceleration, representing a substantial increase from earlier excitations, indicating the onset of significant masonry infill wall dynamic amplification behavior despite the FRPU jacketing. The maximum infill acceleration response of 2.31 g occurred during seismic test number 25 (ST25) at EQ1.4 g earthquake loading intensity, demonstrating severe out-of-plane dynamic response behavior. However, despite the accumulated damage within the mortar bed joint between brick rows that remained uncovered, the high deformability FRPU one-sided jacketing and vertical seismic flexible joints prevented interface debonding (infill-column, or FRPU-brick or FRPU concrete or FRPU—PM joint) or collapse of the out of plane infills. Intrestingly, in-plane partial infill performance was equally successful with no interface debonding, while damage was accumulated mainly at the bottom brick. There, the bottom brick (free infill end) could not sustain compression perpendicular to the brick holes and a toe-compressive failure occurred with disintegration of uncovered internal brick clay walls. The jacketed part of the external clay wall of the bottom brick remained bonded on the FRPU jacket, despite crushed and local FRPU jacket fracture being observed within the same limited region. All these damages ensure human life protection against severe excitations. Time lag analysis between peak foundation acceleration and peak slab acceleration responses revealed temporal delay values ranging from 0.04 s during moderate intensity seismic excitations to -2.88 s during the maximum intensity loading test (negative values indicating slab peak occurring before foundation peak), in other words suggesting complex dynamic interaction and potential localized damage-induced response patterns within the renovated infilled reinforced concrete frame system.

### 3.2. Draw-Wire Results

**Figure 9:**
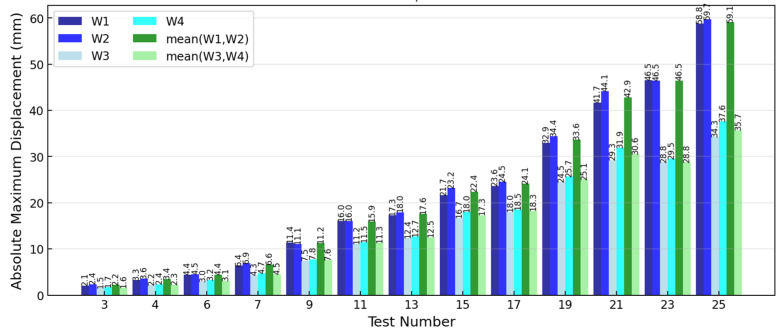
Progressive increase in displacement (seismic load cases).

Draw-wire displacement sensors were used to monitor how the renovated structure moved during testing. The main displacement measurements came from sensors W1-W4. W1 and W2 measured the slab displacement, positioned at the left and right sides, respectively. W3 and W4 measured the foundation beam level displacement at similar positions. When the strongest shaking at EQ1.4 g was executed, the slab showed significant displacement—W1 hit +55.95 mm/−58.84 mm while W2 reached +55.35 mm/−59.73 mm. The foundation in W3 position measured +34.28 mm/−33.79 mm displacement and W4 +34.58 mm/−37.61 mm. A steady increase drift pattern is observed as the EQ intensity increased. The drift started low at 0.8‰ for EQ0.07 g excitation, increased progressively through intermediate stages reaching 11.5‰ at EQ1.1 g, and peaked at 26.2‰ (that is 32.1 mm drift) for the maximum EQ1.4 g excitation. After all excitation, residual drift of −2.37 mm (−1.93‰) was measured, which suggests the structure experienced permanent deformation due to the extreme loading levels and column reinforcement yielding, yet negligible and highly resilient (recovery ability around zero point drift despite very low structure stiffness). Summary of results is depicted in Figure 9 and Table 4.

### 3.3. Strain Gauge Results

During the progressive seismic testing sequence distinct behavioral patterns were observed, with progressive damage development occurring throughout multiple loading stages. During seismic test number 11 (ST11), at EQ0.50 g loading intensity, the opening and closing of horizontal cracks (already formed during Phase A) in brick wall 3-4 was observed without brick wall stiffness reduction. During seismic test number 21 (ST21), at EQ1.3 g loading intensity, several strain gauges demonstrated critical strain magnitudes, with SG5 recording 2693 μstrain, SG6 recording −11803 μstrain (compressive), and SG15 recording 3230 μstrain, coinciding with the columns entering yielding strain of their longitudinal steel reinforcements at the bottom critical regions and the development of crushing of bottom brick at partial infill wall 2-3, at 1.65% structure drift.

During seismic test number 23 (ST23), at the repeated EQ1.3 g (2) loading intensity, damage intensified significantly with crushing of bottom brick at both partial infill walls. Strain gauge SG5 at the external face of column 2 bottom recorded 3511 μstrain tensile strain, SG6 recorded −8657 μstrain compressive strain, and SG15 recorded 1406 μstrain. The maximum recorded strain responses occurred during seismic test number 25 (ST25), at EQ1.4 g loading intensity, with strain gauge SG5 recording the highest tensile strain of 3887 μstrain and compressive strain of 1302 μstrain, SG6 recording 11843 μstrain tensile and −7802 μstrain compressive, indicating that the columns had entered strain hardening of their longitudinal steel reinforcements.

During the structural response analysis of the reinforced concrete elements it was demonstrated that the B500C steel reinforcement exceeded the yield strain threshold of 2890 μstrain during the higher intensity excitations, with maximum measured strains of 3500–3900 μstrain indicating inelastic behavior and confirming column yielding at both top and bottom critical regions. Column 2 of the structure experienced the most severe straining responses, with extreme variations in strain gauge readings from SG6 suggesting complex stress redistribution and potential localized damage effects. During the partial infill wall strain gauge monitoring, significantly lower strain magnitudes were recorded, in other words with maximum values remaining relatively modest despite the severe bottom brick crushing observed, reflecting the different load transfer mechanisms through the PUFJ protected and FRPU jacketed masonry elements of the structure. During the residual strain analysis, substantial permanent strains were revealed in multiple gauges, for EQ1.4 g with SG6 showing residual compressive strain of −4448 μstrain and SG16 showing residual tensile strain of 1415 μstrain, indicating significant cumulative damage effects and permanent deformation while the PUFJ and FRPU systems maintained their integrity. The polyurethane seismic joint strain gauges SG21 and SG22 recorded maximum strains of 1173 μstrain and 803 μstrain, respectively, at EQ1.4 g, confirming that the PUFJ successfully accommodated large tensile deformations without fracture or debonding. The strain gauge measurements provided comprehensive validation of the inelastic performance of the renovated structure at extreme loading levels well beyond the life safety threshold seismic performance level, and confirmed that the observed visual damage to column critical regions and infill walls occurred with column reinforcement yielding while the retrofit systems maintained desirable structural integrity and prevented collapse.

### 3.4. Damage Assessment

The renovated structure was subjected to a comprehensive testing protocol with gradually increasing seismic intensity from EQ0.07 g to EQ1.4 g. Visual inspections were conducted after each excitation to document damage progression in the main structural elements, retrofit interventions, and vertical forest components. No severe damage was observed before the EQ1.1 g excitation, demonstrating the effectiveness of the renovation strategy. The structure ultimately reached extremely high displacement drift levels of 2.62% at EQ1.4 g while maintaining structural integrity and avoiding collapse.

#### 3.4.1. Planter Performance and Anchoring System Behavior

The five concrete planters exhibited varying performance based on their anchoring configurations. Rocking behavior initiated at relatively low excitation levels and progressively intensified throughout the testing sequence, providing valuable insights into the seismic performance of various anchoring strategies.

##### Initial Response (EQ0.07g to EQ0.2g)

**Figure 10:**
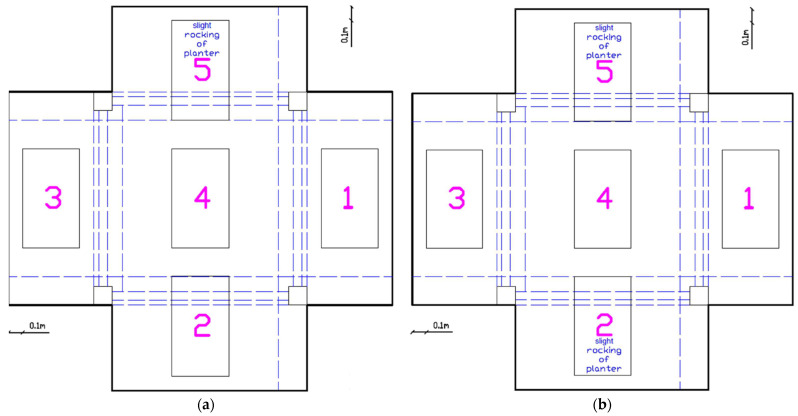
Early-stage behavior up to (**a**) EQ0.1 g slight rocking of planter 2 and (**b**) EQ0.2 g slight rocking of planter 2 and 5.

During the initial excitations up to EQ0.2 g, no damage was observed (Figure 10). Slight rocking of planters 2 and 5 was noticed, indicating that dynamic amplification effects were beginning to affect the roof-mounted elements even at low excitation intensities.

##### Progressive Damage Development (EQ0.34 g to EQ0.55 g)

**Figure 11:**
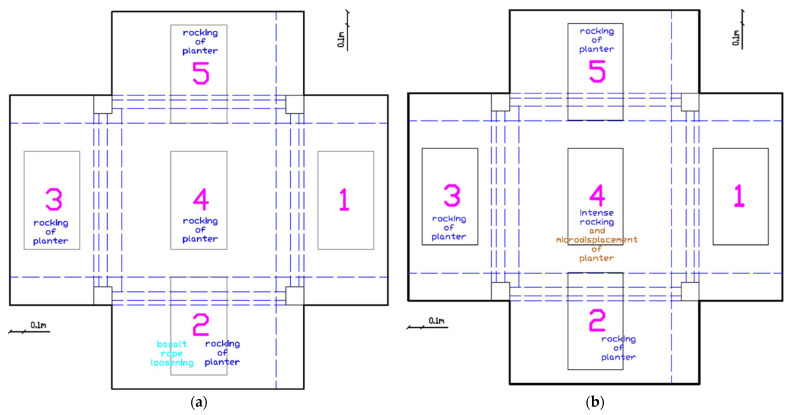

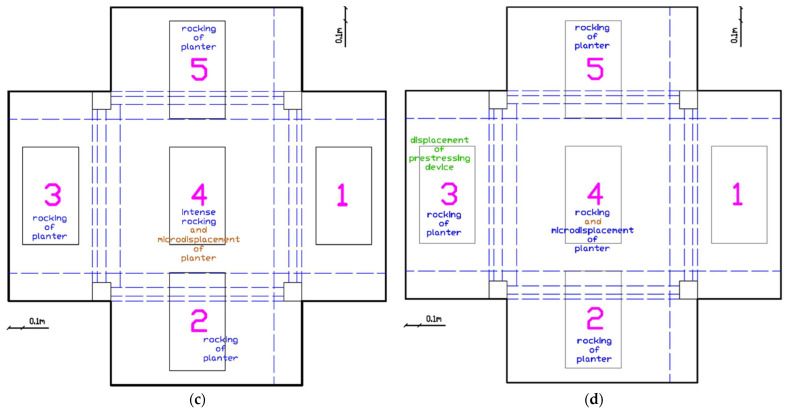
Progressive damage observed at Planters after (**a**) EQ0.34 g, (**b**) EQ0.50 g, (**c**) Wnb0.8 g, and (**d**) EQ0.55.

As seismic intensity increased to EQ0.34 g, more pronounced behavior emerged (Figure 11a). Basalt rope loosening of planter 2 was observed and subsequently tightened. It should be mentioned that in braided rope, the initial post tensioning resulted in stretching of the different strands of the rope, increasing the axial tensile rigidity as the voids among the different strands reduced. However, this loosening suggests that the initial post-tension force was not adequate to eliminate all interstrand voids. Rocking of planters 2, 3, 4, and 5 became evident. At EQ0.5 g, micro-displacement of planter 4 was first noticed (Figure 11b), suggesting that the simply supported configuration on PM pads was beginning to experience sliding. This behavior continued through the EQ0.55 g excitation, with displacement of the prestressing device of planter 3 requiring re-tightening (Figure 11c,d).

##### Severe Damage Initiation (EQ0.74 g to EQ0.8 g)

**Figure 12:**
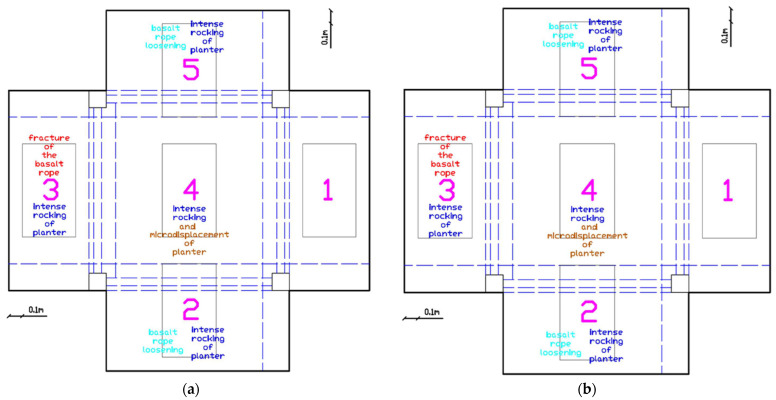
Progressive damage observed at Planters after (**a**) EQ0.74 g, (**b**) EQ0.80 g.

The EQ0.74 g excitation marked a critical threshold where the first basalt rope fracture occurred in planter 3 (Figure 12a). Basalt rope loosening of planters 2 and 5 was also evidenced and the ropes were tightened again. Intense rocking of planters 2, 3, 4, and 5 was observed, indicating significant dynamic amplification at the roof level. After EQ0.8 g, the rocking of planter 4 intensified, leading to micro-displacement of both the PM pads and the planter itself (Figure 12b). The basalt rope of planter 3 continued to loosen despite previous repairs.

##### Advanced Damage State (EQ1.1 g to EQ1.3 g)

**Figure 13:**
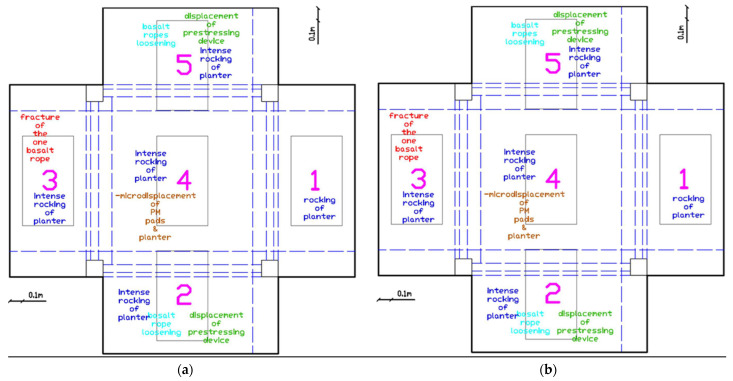
Progressive damage observed at Planters after (**a**) EQ1.1 g, (**b**) EQ1.3 g.

Following the EQ1.1 g excitation, damage accumulation accelerated significantly (Figure 13a). The intense rocking of planter 4 resulted in micro-displacement of the PM pads and the planter itself. One basalt rope of planter 3 fractured completely. Displacement of the prestressing devices of planters 2 and 5 occurred along with loosening of their basalt ropes. Notably, rocking of planter 1 was observed for the first time, suggesting that even the steel rod anchoring system was experiencing increased loading.

The EQ1.3 g excitation produced severe damage in multiple planters (Figure 13b). Planter 4 experienced intense rocking leading to displacement of approximately ±3 cm of both the PM pads and the planter itself. Planter 1 showed bottom micro-displacements due to slight loosening of the steel rod. One basalt rope of planter 2 fractured. Planters 2, 3, and 5 all exhibited bottom displacements, indicating that their anchoring systems were experiencing significant degradation.

##### Maximum Intensity Response (EQ1.3 g (2) to EQ1.4 g)

**Figure 14:**
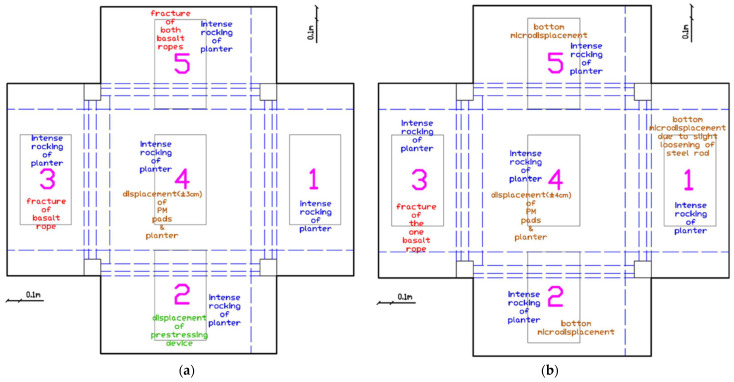
Progressive damage observed at Planters after (**a**) EQ1.3 g (2), (**b**) EQ1.4 g.

After the repeated EQ1.3 g (2) excitation, further deterioration was evident (Figure 14a). The basalt rope of planter 3 fractured, and both basalt ropes of planter 5 fractured completely. The prestressing device of planter 2 was displaced. Despite these severe anchor degradations, no planter collapsed from the roof slab.

At the maximum excitation intensity of EQ1.4 g, planter 4 experienced rocking leading to displacement of approximately ±4 cm of the PM pads and the planter itself (Figure 14b). Planter 1 continued to show bottom micro-displacements due to slight loosening of the steel rod. One basalt rope of planter 3 remained fractured, while planters 2 and 5 showed bottom micro-displacements.

After completion of all excitations, the performance ranking of the five planters was established. Planter 1 (steel rod anchoring through the slab) demonstrated the best overall performance, experiencing only bottom micro-displacements due to slight loosening of the steel rod but maintaining its primary anchoring capacity throughout all excitations. Planter 5 (two fully post-tensioned basalt ropes) showed excellent performance despite eventual rope fracture, with only bottom micro-displacements observed. Planter 2 (drilling through slab with two basalt ropes—one fully post-tensioned and one partially tensioned as safety rope) performed adequately until high excitations when one rope fractured. Planter 3 (two basalt ropes with one fully and one partially post-tensioned) experienced early rope fracture at EQ0.74 g and progressive anchor degradation. Planter 4 (simply supported on PM pads with no basalt anchors) exhibited the poorest performance with significant rocking and displacement initiating at moderate excitation levels and intensifying throughout the testing sequence. Yet, interestingly, up to 0.2 g no rocking, up to 0.34 g only rocking, up to 0.8 g only intense rocking and micro-displacement of planter 4 (no anchoring, only PU pads) was observed without overturning (no planter 1–5 overturned ensuring life-safety).

These results demonstrate that post-tensioned anchoring systems provide superior performance compared to gravity-based support for extreme earthquake excitations, and that steel rod anchoring offers the most reliable connection for heavy roof-mounted elements subjected to severe seismic excitation. Yet, suitably designed flexible, non-corrosive, and natural basalt fiber rope anchors could be a viable, efficient alternative.

#### 3.4.2. Vertical Living Wall Performance

The eight vertical living walls (LW) presented remarkably resilient performance up to very severe seismic excitations. All different attachment configurations using basalt ropes and PU pads sustained high displacement drifts and top slab accelerations. Despite steel rod or basalt rope loosening in some cases at very high accelerations, no living wall collapse was observed throughout the entire testing sequence—a critical finding demonstrating the viability of vertical forest systems for seismic regions.

##### Initial Performance (EQ0.07 g to EQ0.74 g)

No severe loosening of vertical living walls was observed before EQ0.74 g. Minor signs of initiation of loosening of basalt ropes were noticed in LW1-4L and LW3-2L, along with the initiation of micro-loosening in LW1-4R, LW3-2R, LW1-2L, and LW1-2R. However, these early indicators did not compromise structural integrity.

##### Moderate Damage Development (EQ0.8 g to EQ1.1 g)

**Figure 15:**
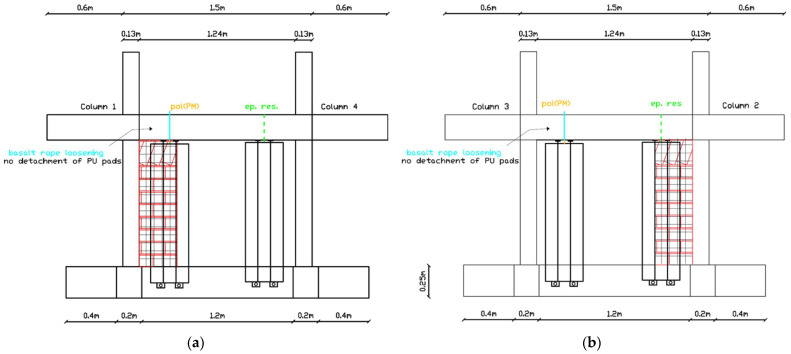
Progressive damage observed at living wall after EQ1.1 g (**a**) LW1-4, (**b**) LW3-2.

After EQ0.8g, left living wall LW1-4L showed basalt rope loosening without detachment of PU pads (Figure 15a). This configuration would allow micro-movements under high direct horizontal loads (severe wind) but maintained adequate resistance for in-plane seismic loading. Similarly, left vertical living wall LW3-2L showed basalt rope loosening without PU pad detachment (Figure 15b). The same damage pattern persisted through the EQ1.1 g excitation for both LW1-4L and LW3-2L.

##### Advanced Damage State (EQ1.3 g to EQ1.3 g (2))

**Figure 16:**
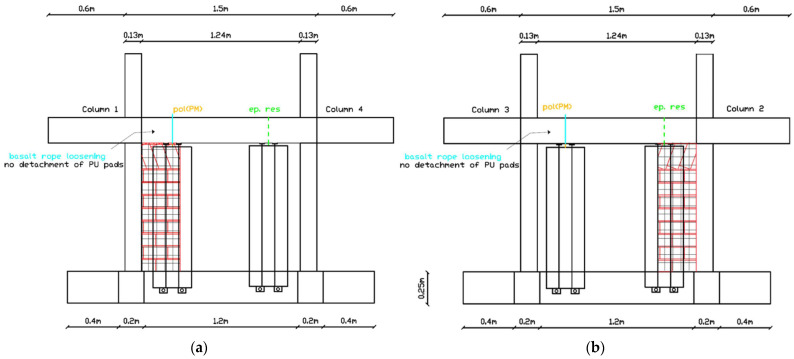
Progressive damage observed at living wall after EQ1.3 g (**a**) LW1-4, (**b**) LW3-2.

Following EQ1.3 g, the damage in LW1-4L and LW3-2L progressed in such a way that micro-movements would be expected under low direct horizontal loads (frequent wind), though still no collapse occurred (Figure 16).

**Figure 17:**
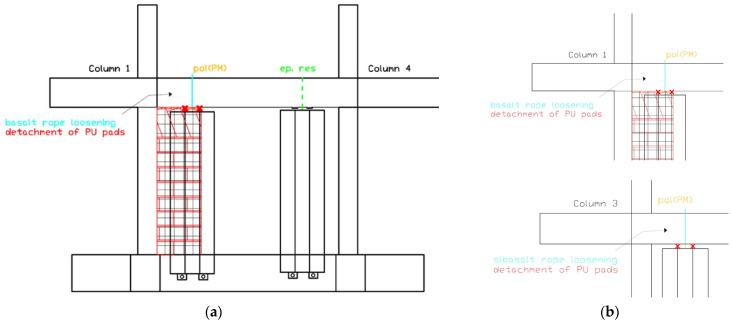

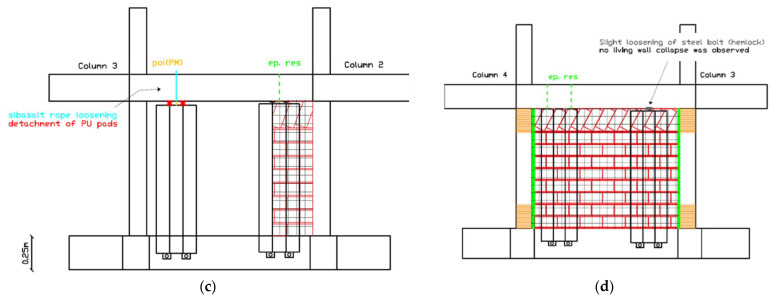
Progressive damage observed at living wall after EQ1.3 g (2) (**a**) LW1-4, (**b**) anchoring details of LW1-4L (top picture) and LW3-2L (bottom picture), (**c**) LW3-2, and (**d**) LW4-3.

The repeated EQ1.3 g (2) excitation marked a critical threshold where basalt rope loosening in LW1-4L and LW3-2L led to detachment of PU pads (Figure 17a–c). This level of damage would allow micro-movements under low direct horizontal loads but the living walls remained attached to the structure. Additionally, steel rod (hemlock) slight loosening was observed at the top of living wall LW3-4R, allowing micro-movements under low horizontal loads (Figure 17d).

##### Maximum Intensity Performance (EQ1.4 g)

**Figure 18:**
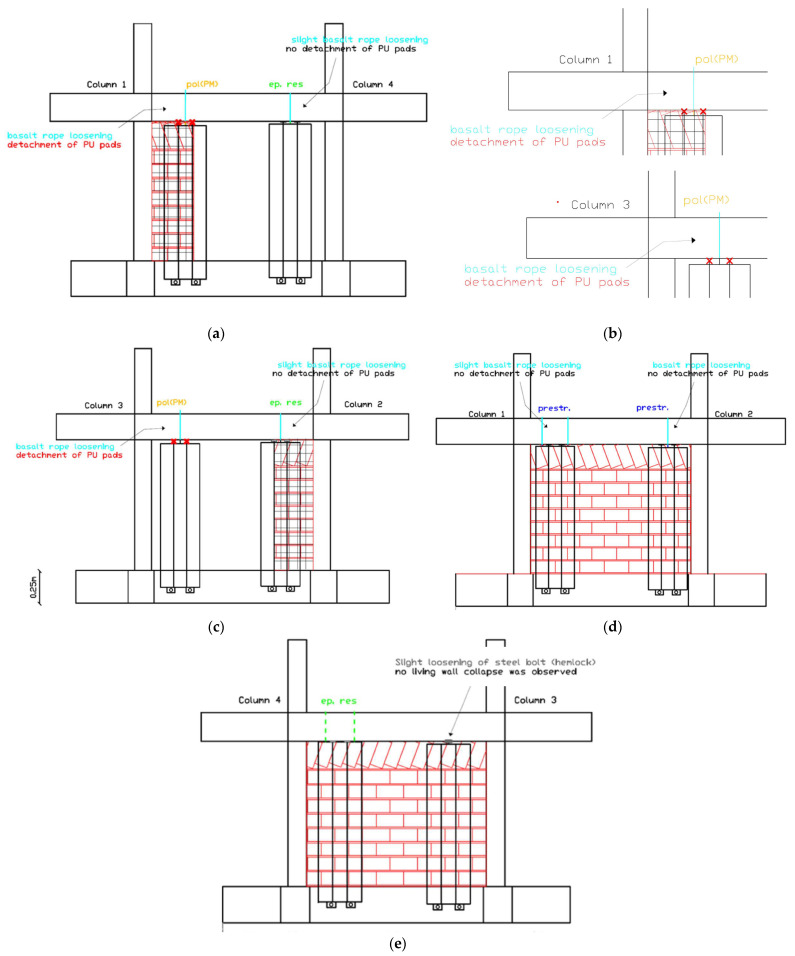
Progressive damage observed at living wall after EQ1.4 g (**a**) LW1-4, (**b**) anchoring details of LW1-4L (top picture) and LW3-2L (bottom picture), (**c**) LW3-2, (**d**) LW1-2 (**e**), LW4-3.

After the maximum intensity excitation of EQ1.4 g, differentiated performance across the eight living walls was clearly established (Figure 18):

- LW3-4L showed the best performance, maintaining full capacity both in-plane and out-of-plane (resistant to severe wind)
- LW1-2L, LW2-3R, and LW1-4R showed slight basalt rope loosening allowing micro-movements for high direct horizontal loads (severe wind) but no collapse
- LW1-2R showed basalt rope loosening allowing micro-movements for medium direct horizontal loads (intense wind) but no collapse
- LW1-4L and LW3-2L showed basalt rope loosening with PU pad detachment, allowing micro-movements for low direct horizontal loads (frequent wind) but no collapse (Figure 18a–d)
- LW3-4R showed steel rod slight loosening allowing micro-movements for low direct horizontal loads but no collapse (Figure 18e)

##### Comparative Living Wall Performance

The results indicate that LW4-3L, LW1-2L, and LW1-2R showed better performance than LW4-3R at very high displacement drifts. Similar high performance was demonstrated by LW1-4L and LW2-3R. The varying behavior suggests that attachment configuration, location on the structure, and loading direction significantly influence vertical living wall seismic performance. Most importantly, the fact that no collapse occurred even after severe anchor degradation demonstrates a robust fail-safe characteristic of the basalt rope—PU pad anchoring system, ensuring life safety.

#### 3.4.3. Main Structural Elements and Retrofit System Performance

##### Damage Progression in Retrofitted Structure

No severe damage to the main structural elements was observed before EQ1.1 g. After EQ1.1 g, cracks with debonding between bricks were noticed in the partial infill walls, indicating that the masonry was beginning to experience distress despite the FRPU jacketing.

##### Column Yielding and Advanced Damage (EQ1.3 g to EQ1.3 g (2))

**Figure 19:**
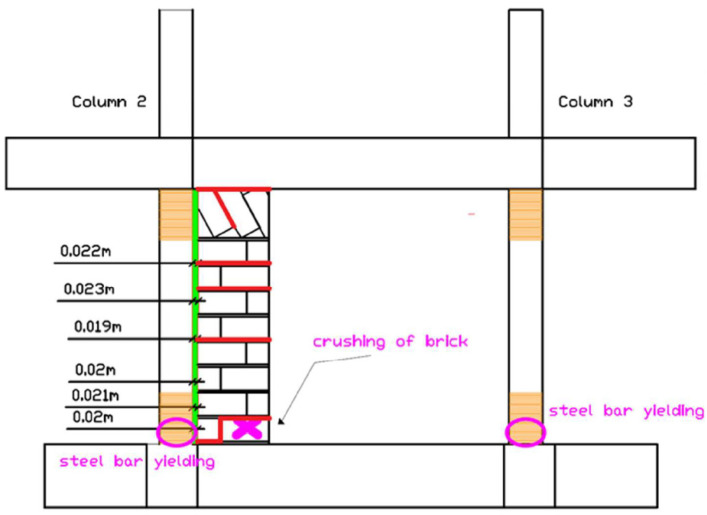
Observed damage after EQ1.3g test: The columns entered yielding strain of their longitudinal steel reinforcements at the bottom. Damage with crushing of bottom brick at partial infill wall 2-3.

The EQ1.3 g excitation marked the transition to advanced damage states (Figure 19). Strain gauge values indicated that the columns entered yielding strain of their longitudinal steel reinforcements at the bottom critical regions. Crushing of the bottom brick at partial infill wall 2-3 was observed, concentrating at the interface with the foundation beam.

**Figure 20:**
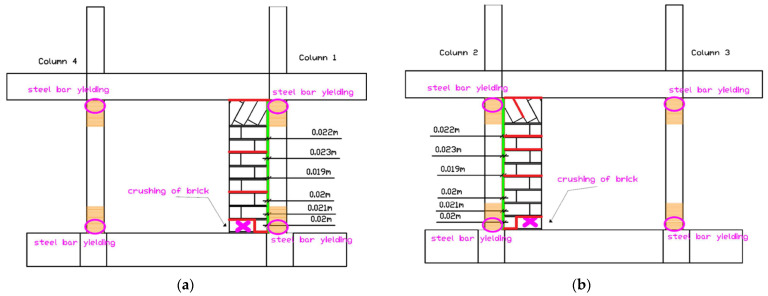
Observed structural damage after EQ1.3 g (2) test at frame (**a**) 4-1 and (**b**) 2-3.

After the repeated EQ1.3 g (2) excitation, damage intensified significantly (Figure 20). Crushing of the bottom brick occurred at both partial infill walls 1-4 and 2-3. The columns entered yielding strain of their longitudinal steel reinforcements at both the bottom and top critical regions (columns 1, 2, 3, and 4), according to strain gauge measurements. Despite these severe damage states, the PUFJ between concrete columns and brick infills maintained their integrity without fracture or debonding. The FRPU jackets maintained their bonding on the PUFJs, on the concrete, and on the bricks. No failure of brick infills 1-2 and 4-3 (loaded out-of-plane) was observed, demonstrating the effectiveness of the retrofit in preventing infill collapse.

##### Ultimate Damage State (EQ1.4 g)

**Figure 21:**
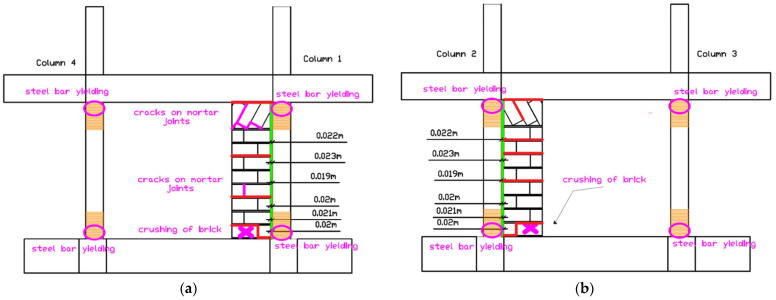

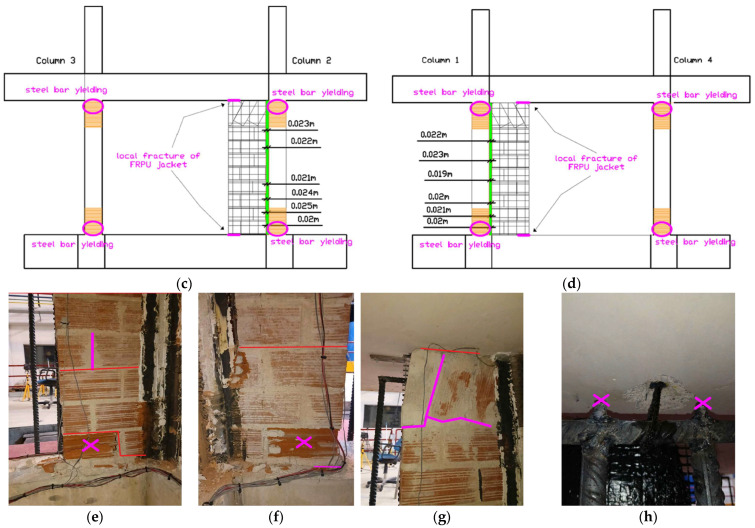
Observed structural damage after EQ1.4 g test at frame (**a**) 4-1, (**b**) 2-3, (**c**) 3-2, (**d**) 1-4, and characteristic observed damage after all seismic tests: (**e**) interior side of wall infill 1–4 parallel to the direction of movement of seismic table, (**f**) wall infill 2–3 parallel to the direction of movement of seismic table, (**g**) interior top side of wall infill 1–4, parallel to the direction of movement of the seismic table, (**h**) detachment of PU pads.

After the maximum intensity excitation of EQ1.4 g at extremely high displacement drift levels of 2.62%, the ultimate damage pattern was established (Figure 21a–d, and characteristic damage in Figure 21e). Damage with the crushing of the bottom brick at both partial infill walls occurred at the interface with the foundation beam. Local fracture of the FRPU jacket at the boundary between the crushed brick and the bottom foundation beam was observed, representing the only failure of the retrofit system and occurring only at the extreme localized crushing zone. Additional cracks developed at some mortar joints between bricks of the partial infill walls at their internal face (the side with no FRPU jacket). The columns remained in the yielded state at both bottom and top critical regions.

#### 3.4.4. Overall Structural Performance Assessment

The second phase of seismic testing of the VF renovated structure was completed successfully after the execution of 28 dynamic tests. The innovative renovation strategy avoided partial or global collapse of the structure. At the end of all tests, the structure showed extremely low stiffness around zero displacement drift, indicating severe damage accumulation and yielding of the primary structural elements. However, the structure revealed recovery potential with negligible residual drift, suggesting that the retrofit system maintained sufficient integrity to allow the structure to return toward its original position after load removal, that is, advanced resilience characteristics with exceptional insurance of life-safety, desirable damage hierarchy, and potential for re-strengthening.

The PUFJ maintained integrity throughout all excitations without fracture or debonding, successfully accommodating the large differential movements between the concrete frame and brick infills. The FRPU jackets maintained their bonding on the PUFJs, concrete, and bricks throughout the testing sequence, failing only locally at the extreme crushing zone where bottom bricks were completely crushed. The out-of-plane infill walls (1-2 and 4-3) showed no failure, validating the retrofit effectiveness for preventing infill collapse under bidirectional loading. The basalt rope confinement at column critical regions contributed to maintaining column integrity despite longitudinal reinforcement yielding, with no basalt fracture or loosening or concrete core bulging.

### 3.5. Assessment of the Structure Using Advanced PZTs Results

Real-time structural health monitoring throughout the seismic testing was achieved using a wireless system based on the fundamental principles of the EMI method [28,29]. This system utilizes the advantageous properties of the piezoelectric phenomenon and PZT sensors to detect any irregularities in the structural elements. The system exploits the inverse piezoelectric effect, enabling the PZT sensors to function as both actuators and sensors simultaneously. When subjected to alternating electrical oscillations, a PZT patch—whether epoxy-bonded to or embedded within a structural component—generates high-frequency mechanical oscillations. These oscillations are contingent upon the local material characteristics, including stiffness, mass, and damping [30,31]. According to Naoum et al., embedded PZT sensors, like smart aggregates, are more sensitive to damage development and stress distributions within the host structure than externally bonded PZT patches [17]. The electrical signal generated by the sensor directly correlates with the mechanical impedance of the monitored region. Structural deterioration mechanisms such as cracking, debonding, or material degradation modify these local mechanical characteristics, which in turn alter the impedance signature captured by the PZT [32,33]. This electromechanical coupling enables damage to be identified at an early stage, typically well before any visual evidence emerges.

Recent research has demonstrated the effectiveness of the EMI method in assessing deterioration of structural integrity caused by various factors, such as corrosion of reinforcement in reinforced concrete elements, heating time, and loss of concrete mass [34,35]. Additionally, researchers have examined the EMI method’s effectiveness in detecting various types of structural damage, including cracks in concrete, steel reinforcement deformation, and stress level assessment [36,37,38]. Furthermore, numerous research articles have investigated identifying the location and severity level of damage to standard concrete specimens under different induced loading procedures [18,39,40,41].

For Phase A, 48 PZT sensors were strategically placed at critical structural zones, covering columns, beam-column joints, beams, slabs, cantilevers, and brick infill surfaces. In Phase B, the monitoring system was expanded to assess the performance of the retrofit interventions and the structural impact of the vertical forest attachments. More specifically, 13 additional PZTs were installed on the renovated structure to monitor the health of the retrofits and the connection (anchoring) of the vertical living wall and planter constructions, bringing the total to 61 PZT sensors for evaluating the renovated frame structure. The sensors recorded electromechanical impedance data within a 10–250 kHz frequency band at 1 kHz intervals, producing voltage signals representing local structural conditions.

For Phase A, reference signatures were obtained from the undamaged structure prior to seismic testing. For Phase B, the healthy pristine state was established after the completion of all renovations—including basalt rope wrapping, PUFJ installation, FRPU jacketing, and vertical living wall or planter attachment—but before any seismic table excitations of the second phase. This baseline served as the reference for subsequent damage evaluation throughout the testing sequence, presented in this study.

Damage quantification utilized the Root Mean Square Deviation (RMSD) metric, which is considered one of the most effective and reliable indices in Structural Health Monitoring (SHM) applications. The RMSD translates variations in EMI signatures into numerical values, comparing baseline and post-damage measurements:

(1) $$RMSD=\sqrt{\frac{\sum_{i=1}^{M}{\left({\left|V_{p}\left(f_{i}\right)\right|}_{D}-{\left|V_{p}\left(f_{i}\right)\right|}_{0}\right)}^{2}}{\sum_{i=1}^{M}{\left({\left|V_{p}\left(f_{i}\right)\right|}_{0}\right)}^{2}}}$$

where |Vₚ(fᵢ)|_0_ represents the voltage signal from the PZT at the undamaged baseline state, |Vₚ(fᵢ)|_D_ represents the voltage signal at any damaged condition, M indicates the total measurement points in the frequency range, and i denotes individual frequency increments. Elevated RMSD values correspond to more significant structural modifications and damage severity, offering a quantifiable assessment metric. It was observed that elevated RMSD values were not present in PZTs situated in regions where no cracking was detected, confirming the reliability of the index for damage localization and assessment.

RMSD values were calculated from the absolute voltage output responses to assess the effectiveness of the SHM method in damage detection and evaluation throughout Phase B testing. The analysis compared voltage frequency responses at various excitation levels (EQ0.1 g, EQ0.5 , EQ0.8 g, EQ1.1 g, EQ1.3 g, and the final EQ0.2 g excitation) against the healthy pristine baseline state established after all renovations were completed. A series of measurements were performed on each PZT sensor in a healthy state to determine a baseline frequency response. During the seismic loading process, three consecutive measurements were taken to improve the reliability and accuracy of the method. Any extreme values observed, possibly due to noise, were reviewed and removed. The acquired EMI responses from the PZTs were automatically uploaded by the measuring devices into the database for post-processing, and the EMI procedure was performed on one measurement from each seismic excitation. The results demonstrate the effectiveness and reliability of the implemented instrumentation and the adopted EMI-based monitoring method for structural health assessment of the renovated vertical forest structure.

#### 3.5.1. Damage Assessment by Structural Component

##### Beams and Foundation Elements

**Figure 22:**
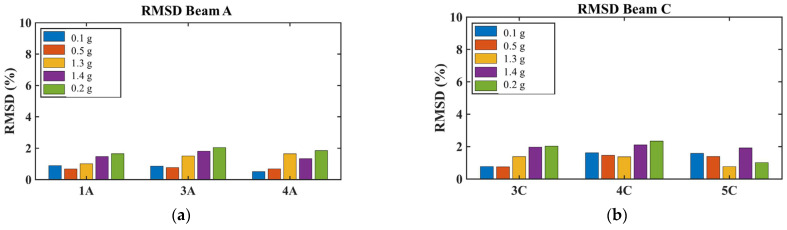
RMSD values from voltage frequency responses of the PZT sensors inside (**a**) beam B14 and (**b**) beam B12.

No significant values were observed in the RMSD indices for PZT sensors installed on the beams, where no structural damage was detected (Figure 22). However, there is a trend of increasing RMSD values that could trigger further actions to assess potential internal micro-damages in the beams. This gradual increase suggests that while the beams remained largely undamaged, minor internal deterioration may have begun to develop during the more intense excitations.

##### Slab Response

**Figure 23:**
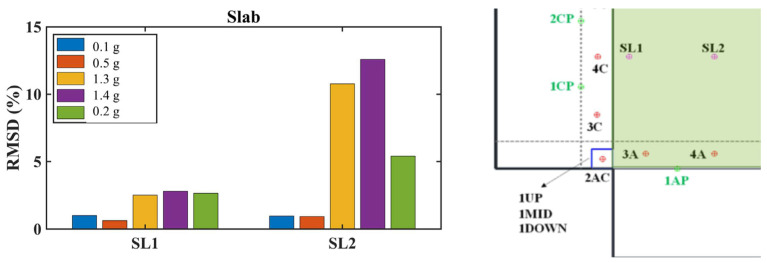
RMSD values from voltage frequency responses of the PZT sensors inside the slab.

As mentioned above, PZT sensors were installed in meticulously selected positions on the slab to enable the prompt detection of potential damage in this area. The PZT sensors exhibited different behavior depending on their location on the slab (see Figure 23). Specifically, the PZT SL2 sensor was installed in the central region of the slab, as a smart aggregate under the central planter (Planter 4). According to the experimental design, the central planter lacked anchoring and exhibited intense rocking during the shaking tests. The increased RMSD values in the central area (PZT SL2) demonstrate the EMI method’s ability to capture the accumulation of damage in the mid-section of the RC slab. Specifically, the intense vibration of Planter 4 during seismic excitation elevates the RMSD values of PZT SL2 after EQ1.3 g. In contrast, the PZT sensors located at the edge of the slab exhibited lower RMSD values, indicating that they experienced less severe damage compared to those in the central area where planters were installed.

##### Column Performance and Retrofit Effectiveness

**Figure 24:**
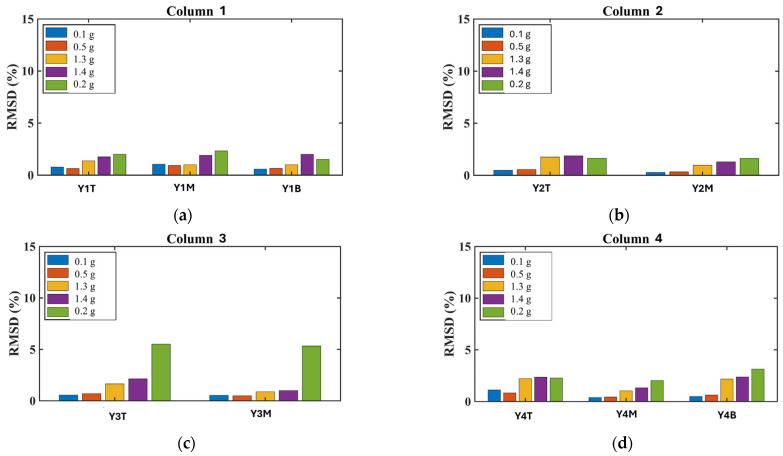
RMSD values from voltage frequency responses of the PZT sensors inside (**a**) column 1 (top, middle, bottom), (**b**) column 2 (top, middle), (**c**) column 3 (top, middle), and (**d**) column 4 (top, middle, bottom).

All columns showed increased RMSD values at all monitored regions (bottom, middle, and top), indicating that increasing additional damage had developed within the concrete core beyond the damage sustained during Phase A (Figure 24). After the EQ1.3 g excitation, all columns exhibited higher RMSD values compared to the EQ0.1 g and EQ0.5 g excitations. Following the EQ1.3 g excitation, the PZT sensors recorded higher RMSD values that showed an increasing trend continuing through the subsequent EQ1.3 g and final EQ0.2 g excitations, especially in column 3.

Comparative analysis between columns revealed important insights into retrofit effectiveness. Relatively lower RMSD values in columns 1 and 2 compared with those in columns 3 and 4 may indicate that the basalt rope wrapping together with PUFJ and FRPU jacketed infills in two directions (both full and partial infills adjacent to columns 1 and 2) could restrict additional concrete core disintegration within the critical regions more effectively than in columns 3 and 4, which only had full infill in one direction. This observation suggests that the combination of multiple retrofit interventions and infill configurations in perpendicular directions provided enhanced protection against progressive damage.

##### Infill Wall Behavior

**Figure 25:**
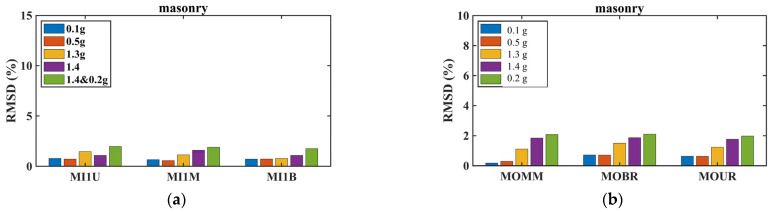
RMSD values from voltage frequency responses of the PZT sensors (**a**) on partially infilled walls between column 1-4 and (**b**) on full infill wall between columns 1-2.

Slight differences were observed in the RMSD values of PZTs installed on the surfaces of the partial infill walls and the full infill wall within the polyurethane joint (Figure 25 PZT sensors were installed during phase A and are shown before FRPU jacketing.). After the EQ1.3 g excitation, the RMSD values were higher compared to the EQ0.1 g and EQ0.5 g excitations, indicating progressive damage development in the infill elements as seismic intensity increased.

#### 3.5.2. Retrofit Component Monitoring

##### Column Surface and Interface Monitoring

**Figure 26:**
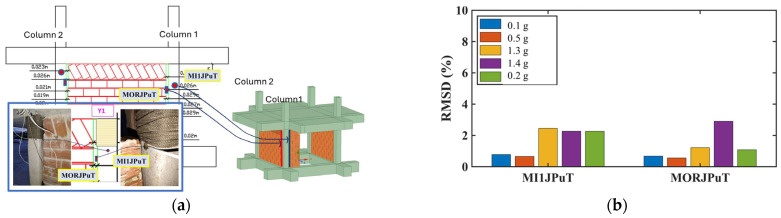
(a) Positions of the PZT sensors, (b) RMSD values from voltage frequency responses of the PZT sensors placed on top of column 1: MORJPuT at the infill wall and MI1JPuT at the partial infill wall.

PZT patches installed at the surface of RC column 1, including MORJPuT at the infill wall and MI1JPuT at the partial infill wall, provided valuable information about interface behavior (Figure 26). Elevated RMSD values were recorded at the PZTs externally attached on the surface of column 1 at the top, particularly for the partial infill wall experiencing in-plane loading. This suggests that the column–infill interface experienced increased stress and potential local damage progression during more intense excitations (probably coupled with the concrete damage inside the critical region of the column).

##### Vertical Living Wall Anchoring System

**Figure 27:**
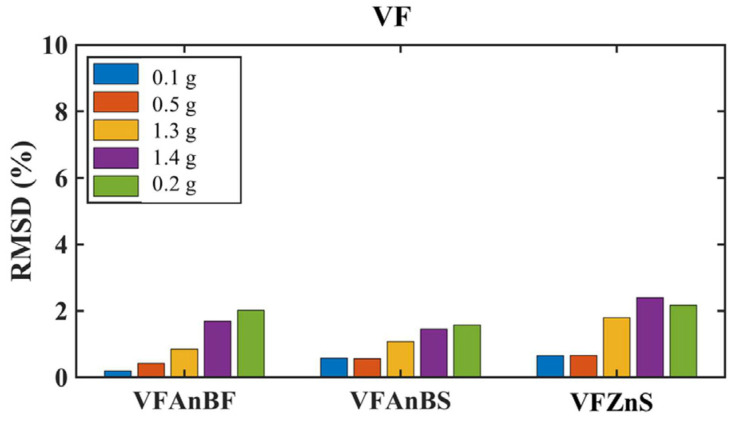
RMSD values from voltage frequency responses of the PZT sensors (column 2: VFAnBF, LW2-3R, column 4: VFAnBS, LW1-4R anchoring metal structure for living wall as well as anchoring metal structures for planter 1, VFZnS).

PZT patches externally attached to the basalt ropes at the top of the living walls, anchored inside the top slab using resin, showed no significant changes in voltage frequency response and exhibited relatively low RMSD values while capturing the increasing trend with increasing seismic intensity (Figure 27). The recordings from living walls LW2-3R (VFAnBF, close to column 2) and LW1-4R (VFAnBS, close to column 4) align with the observation that no collapse of these vertical living wall constructions occurred, although the basalt ropes experienced higher tension during more intense seismic excitations. This demonstrates that the anchoring system performed well throughout the testing sequence.

##### Planter Anchoring Performance

PZT patches installed on the anchoring metal structure of planter 1 (VFZnS) exhibited elevated RMSD values after the EQ1.3g excitation (Figure 27). This increase agrees with the slight loosening of the steel rod anchoring system observed during visual inspection, confirming the sensor’s ability to detect negligible deterioration in anchoring performance.

##### Basalt Rope Confinement Monitoring

**Figure 28:**
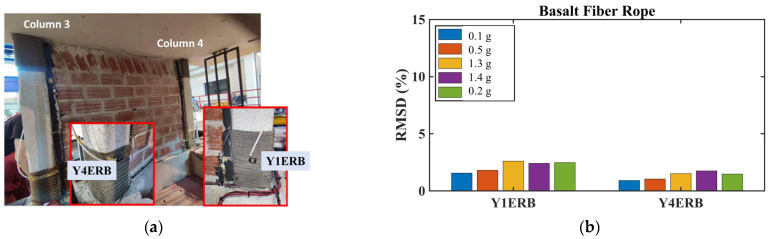
(a) Positions of the PZT sensors, (b) RMSD values from voltage frequency responses of the PZT sensors externally attached (Y4ERB and Y1ERB—columns 3 and 4 bottom) on the basalt rope.

PZT patches installed on the basalt rope wrapping of columns 3 and 4 exhibited different voltage frequency responses compared to the usual patterns observed in concrete elements (Figure 28).

While the efficacy of the EMI method for SHM in reinforced concrete structural elements has been thoroughly examined in the literature, a notable research gap exists concerning its investigation for composite materials such as basalt fiber systems. The PZT recordings captured the increasing trend of basalt tightening despite the extremely low strain values measured in the basalt ropes. This demonstrates the high sensitivity of the EMI method for monitoring composite confinement systems, even when strains remain within elastic ranges.

The comprehensive PZT monitoring system successfully tracked damage progression throughout the Phase B testing sequence. Key findings include the following:

- Damage localization capability: Elevated RMSD values corresponded accurately with visually observed damage locations, while sensors in undamaged regions maintained low values.
- Retrofit effectiveness quantification: Lower RMSD values in columns with combined retrofit strategies (basalt rope + PUFJ + FRPU with bidirectional infills) suggest enhanced damage resistance compared to columns with single-direction infill configurations.
- Early warning potential: Progressive increases in RMSD values provided advance indication of damage accumulation before severe deterioration observations.
- Composite material monitoring: The method proved sensitive enough to monitor basalt rope confinement systems, extending its applicability beyond conventional concrete elements.
- Anchoring system assessment: PZT sensors successfully detected deterioration in vertical forest anchoring systems, demonstrating their potential in monitoring novel structural connections.

These results support the effectiveness and reliability of the implemented instrumentation and confirm that the EMI-based monitoring method provides valuable real-time structural health assessment for renovated vertical forest structures subjected to seismic loading.

## 4. Conclusions

The second phase of seismic testing was completed successfully after 28 dynamic tests with peak ground acceleration ranging from EQ0.07 g to EQ1.4 g, employing a methodological approach of gradually increased intensity similar to a dynamic pushover approach. The innovative renovation strategy successfully prevented partial or global infill or RC structure or VLW or planter collapse despite extremely demanding loading conditions. At maximum EQ intensity the structure experienced drift levels of 2.62% with negligible residual drift of −1.93‰, suggesting that the retrofit system provided sufficient integrity to allow the structure to return toward its original position after load removal. That is, despite extremely low structure global stiffness, it presents advanced global resilience characteristics and potential for re-strengthening.

The structural retrofit interventions exhibited excellent performance. The highly flexible PUFJ maintained integrity throughout all excitations without fracture or debonding, successfully accommodating large differential movements. The FRPU system maintained bonding throughout testing, failing only locally at the extreme crushing zone where bottom bricks were completely crushed. The reused, high temperature-resistant, reusable, recyclable, natural, and corrosion-resistant flexible basalt fiber rope confinement at column critical regions contributed to column integrity despite longitudinal reinforcement entering yielding and strain hardening. Column 2 experienced the most severe straining responses with SG6 recording 11,843 μstrain tensile, while residual compressive strains of −4448 μstrain confirmed significant cumulative damage effects in the concrete core of critical regions.

The eight vertical living wall constructions presented remarkably resilient performance demonstrating the viability of vertical forest systems for seismic regions. Despite basalt rope loosening and PU pad detachment in LW1-4L and LW3-2L after EQ1.3 g (2), no living wall collapse was observed throughout the entire testing sequence, demonstrating a robust fail-safe characteristic where even severe anchor degradation did not result in complete detachment.

The five concrete planters exhibited varying performance based on anchoring configurations. Planter 1 with steel rod anchoring demonstrated the best overall performance maintaining primary anchoring capacity throughout all excitations, planter 5 with two fully post-tensioned basalt ropes showed excellent performance despite eventual rope fracture, while planter 4 simply supported on PM pads exhibited base displacement of ±4 cm at maximum intensity, yet avoiding planter overturning in all cases despite intense rocking behavior. These results demonstrate that post-tensioned anchoring systems provide superior performance compared to gravity-based support.

The comprehensive PZT monitoring system (61 sensors total) utilizing the EMI technique successfully tracked damage progression throughout testing. Elevated RMSD values corresponded accurately with visually observed damage locations; in other words, relatively lower RMSD values in columns 1 and 2 compared with columns 3 and 4 indicated that basalt rope wrapping together with PUFJ and FRPU jacketed infills in two directions could restrict additional concrete core disintegration more effectively. The method proved sensitive enough to monitor both conventional concrete elements and composite retrofit materials; notably, PZT recordings captured the increasing trend of basalt tightening despite extremely low strain values, demonstrating high sensitivity for monitoring composite confinement systems.

The results demonstrate that properly designed vertical forest renovations can achieve satisfactory seismic performance even under extreme loading conditions that significantly exceed typical design basis earthquake demands. The combination of strategic structural retrofitting with appropriately anchored greenery interventions provides a viable pathway for sustainable urban development that integrates green, natural, reusable, recyclable, energy saving, corrosion- and elevated temperature-resistant, and advanced human comfort hybrid envelop infrastructure (composites and greenery) with resilient seismic design.

This research advances sustainable infrastructure renovations in general by validating the integration of vertical forest systems with natural, recyclable composite materials on seismically deficient existing building stock. The basalt fiber rope confinement (reused from previous studies, demonstrating reusability) system proved effective without epoxy resins. Most critically, the experiments confirm that older Mediterranean RC buildings can safely support vertical forest loads (795 kg planters + 96 kg VLW systems on a single-story frame, scalable to multi-story applications) even under extreme seismic excitation (2.62% drift, 1.4 g PGA). Despite severe anchor degradation in some cases, no planter or VLW collapse occurred, ensuring life safety while maintaining the protective green building envelope. The validated anchoring strategies and monitoring methodology provide the basic technical foundation for supporting widespread implementation of vertical forest renovation—enabling existing structures to simultaneously enhance seismic resilience, sequester carbon, improve energy performance, and mitigate urban heat island effects without demolition and reconstruction. This integrated approach demonstrates that seismic safety and environmental sustainability are complementary rather than competing objectives in building stock upgrades.

## Figures and Tables

**Table 1:** Column confinement basalt fiber rope details.

Column	Side	Inside Layer Spirals	Outside Layer Spirals	Spirals per Side	Total Spirals
1	Bottom	53	11	64	127
	Top	50	13	63	
2	Bottom	48	15	63	124
	Top	50	11	61	
3	Bottom	48	18	66	135
	Top	49	20	69	
4	Bottom	45	30	75	143
	Top	48	20	68	

**Table 2:** Total Test Sequence protocol for Phase B (28 tests).

Test No.	Name	Type	Intensity	Test No.	Name	Type	Intensity
ST1	WNb0.07 g (without)	White noise	0.05 g	ST15	EQ0.74 g	Earthquake	0.74 g
ST2	WNb0.07 g	White noise	0.05 g	ST16	WNb0.8 g	White noise	0.08 g
ST3	EQ0.07 g	Earthquake	0.07 g	ST17	EQ0.8 g	Earthquake	0.80 g
ST4	EQ0.1 g	Earthquake	0.10 g	ST18	WNb1.1 g	White noise	0.08 g
ST5	WNb0.14 g	White noise	0.08 g	ST19	EQ1.1 g	Earthquake	1.10 g
ST6	EQ0.14 g	Earthquake	0.14 g	ST20	WNb1.3 g	White noise	0.08 g
ST7	EQ0.2 g	Earthquake	0.20 g	ST21	EQ1.3 g	Earthquake	1.30 g
ST8	WNb0.34 g	White noise	0.08 g	ST22	WNb1.3 g (2)	White noise	0.08 g
ST9	EQ0.34 g	Earthquake	0.34 g	ST23	EQ1.3 g (2)	Earthquake	1.30 g
ST10	WNb0.5 g	White noise	0.08 g	ST24	Wnb1.4 g	White noise	0.08 g
ST11	EQ0.5 g	Earthquake	0.50 g	ST25	EQ1.4 g	Earthquake	1.40 g
ST12	WNb0.55 g	White noise	0.08 g	ST26	Wnb0.2 g (2)	White noise	0.08 g
ST13	EQ0.55 g	Earthquake	0.55 g	ST27	EQ0.2 g (2)	Earthquake	0.20 g
ST14	WNb0.74 g	White noise	0.08 g	ST28	Wna0.2 g	White noise	0.08 g

**Table 3:** Summary of results for accelerations (28 total tests).

Test No.	PGA (g)	PFA (g)	FAF	Test No.	PGA (g)	PFA (g)	FAF
ST1	0.04	0.07	1.75	ST15	0.72	1.33	1.85
ST2	0.04	0.09	2.25	ST16	0.07	0.16	2.29
ST3	0.07	0.12	1.71	ST17	0.88	1.31	1.49
ST4	0.09	0.18	2.00	ST18	0.07	0.22	3.14
ST5	0.06	0.197	3.28	ST19	1.33	1.66	1.24
ST6	0.14	0.22	1.57	ST20	0.07	0.18	2.57
ST7	0.21	0.36	1.71	ST21	n.a.	n.a.	n.a.
ST8	0.08	0.19	2.38	ST22	n.a.	n.a.	n.a.
ST9	0.36	0.53	1.47	ST23	1.87	1.80	0.96
ST10	0.08	0.17	2.13	ST24	0.06	0.19	3.17
ST11	0.58	0.77	1.33	ST25	2.56	1.80	0.70
ST12	0.07	0.18	2.57	ST26	0.05	0.19	3.80
ST13	0.63	0.91	1.44	ST27	0.19	0.37	1.95
ST14	0.07	0.19	2.71	ST28	0.05	0.2	4.00

n.a. = not available.

**Table 4:** Summary of results for relative bottom-top displacements (drift for 28 total tests).

Test No.	Disp. (mm)	Drift (‰)	Test No.	Disp. (mm)	Drift (‰)
ST1	0.8	0.7	ST15	10.5	8.6
ST2	0.8	0.7	ST16	1.7	1.4
ST3	1	0.8	ST17	11.2	9.1
ST4	1.5	1.2	ST18	2.6	2.1
ST5	1.8	1.5	ST19	14.1	11.5
ST6	1.9	1.6	ST20	2.4	2.0
ST7	2.8	2.3	ST21	20.2	16.5
ST8	1.7	1.4	ST22	3.9	3.2
ST9	5.6	4.6	ST23	24.6	20.1
ST10	1.7	1.4	ST24	6.6	5.4
ST11	6.8	5.6	ST25	32.1	26.2
ST12	1.7	1.4	ST26	9.5	7.8
ST13	8.4	6.9	ST27	12.5	10.2
ST14	1.9	1.6	ST28	9.6	7.8

## Data Availability

The original contributions presented in this study are included in the article. Further inquiries can be directed to the corresponding authors.
